# Eveningness in Middle‐Aged and Older Adults: Associations With Sleep, Internalising Symptoms, and Alertness

**DOI:** 10.1111/jsr.70104

**Published:** 2025-06-02

**Authors:** Xinran Niu, Kristin E. G. Sanders, Elizabeth A. Kensinger, Jessica D. Payne

**Affiliations:** ^1^ Department of Psychology University of Notre Dame Notre Dame Indiana USA; ^2^ Department of Psychology and Neuroscience Boston College Chestnut Hill Massachusetts USA

**Keywords:** aging, alertness, anxiety, chronotype, depression, sleep

## Abstract

Evening chronotype, characterised by a preference for later circadian timing, can present challenges in societal activities oriented towards morning schedules. This study investigates the link between chronotype and sleep, alertness, and internalising symptoms in middle‐aged and older adults. The sample comprised 652 healthy adults (ages 35–98) free of sleep, psychiatric, or neurological disorders. All participants completed a 3‐min version of the Psychomotor Vigilance Test both in the morning and evening, where the median reaction time served as a measure of behavioural alertness. Additionally, participants completed: (1) the Morningness–Eveningness Questionnaire for chronotype, (2) the Stanford Sleepiness Scale for subjective alertness, (3) the Pittsburgh Sleep Quality Index for sleep disturbance, and (4) the Mood and Anxiety Symptom Questionnaire for depression‐specific anhedonia, anxiety‐specific anxious arousal, and the common internalising factor, general distress. Results indicated that endorsing an evening chronotype provided a transdiagnostic risk factor for general distress, anhedonia, anxious arousal, as well as sleep disturbance and morning subjective sleepiness across middle‐aged and older adults. Evening chronotypes showed higher behavioural alertness in the evening, compared to both intermediate and morning chronotypes. These results suggest that chronotype should be considered when promoting healthy ageing and optimal performance.

## 1 Introduction

Chronotype refers to an individual's internal preferences for sleep–wake timing (Roenneberg et al. 2019). Chronotype manifests as a spectrum, with “morningness” characterised by an earlier sleep onset and offset, and a peak in physical and mental performance earlier in the day, and “eveningness” defined by a preference for later sleep and rise times and better performance in the evening (Roenneberg et al. 2019). Individual differences in chronotype arise from a range of physiological factors, such as body temperature, cortisol release, and melatonin rhythm throughout the day (Adan et al. 2012; Hood and Amir 2017). Misalignment between internal chronotype and external factors (e.g., traditional work start times, light exposure) disrupts circadian rhythms, leading to social jetlag, or the discrepancy between an individual's internal sleep–wake cycle and external demands (Roenneberg et al. 2019). Therefore, evening chronotypes often struggle with typical early morning work/school schedules (Roenneberg et al. 2019).

In general, compared to morningness, eveningness is associated with worse outcomes in domains of physical health (Suh et al. 2017), psychological well‐being (Norbury 2021; Zou et al. 2022) and cognitive functioning (Schmidt et al. 2012). This is due to, at least in part, reduced physical activity and exposure to natural light, which are critical factors in regulating sleep–wake cycles (Böhmer et al. 2021). These disruptions in sleep–wake cycles can further accumulate sleep pressure related to social jetlag, exacerbate poor sleep quality, and negatively impact daytime alertness (Niu et al. 2021; Roenneberg et al. 2019). Disruptions in circadian alignment and sleep quality are also linked to more severe symptoms of depression and anxiety (Niu et al. 2021; Niu and Snyder 2022).

Prior studies consistently link eveningness to an increased risk of depression and anxiety (Norbury 2021; Zou et al. 2022). However, given the high comorbidity between depression and anxiety, chronotype may not serve as an independent predictor for depression and anxiety (Hankin et al. 2016). Recent studies advocate for depression and anxiety as latent dimensions of internalising symptoms rather than discrete psychiatric disorders (Hankin et al. 2016). This perspective highlights the need to determine whether eveningness exerts similar or distinct effects on symptoms specific to depression, specific to anxiety, and those shared between depression and anxiety. Properly distinguishing these dimensions is essential for developing circadian‐informed interventions tailored to specific forms of internalising psychopathology.

This gap in understanding the specific ways in which chronotype influences different dimensions of internalising symptomatology is particularly important when considering the natural shifts in chronotype across the lifespan. Chronotype shifts from morningness in childhood to eveningness in young age to morningness again in late adulthood (Fischer et al. 2017). A phase‐delayed evening chronotype may be particularly detrimental for older adults, impacting their cognitive performance, overall health, and longevity (Didikoglu et al. 2019; Schmidt et al. 2012; Suh et al. 2017). This is likely because older adults typically experience a weakened endogenous control over their circadian rhythms (Didikoglu et al. 2019; Hood and Amir 2017; Kim et al. 2022). As a result, older adults often face greater difficulties recovering from the sleep disruption caused by social jetlag and experience increased daytime sleepiness and internalising symptoms (Kim et al. 2022; Sinha et al. 2023). Previous research has primarily examined the negative consequences of eveningness in older adults (Didikoglu et al. 2019; Hood and Amir 2017; Kim et al. 2022; Schmidt et al. 2012; Suh et al. 2017), with fewer studies including middle‐aged adults (e.g., Kianersi et al. 2023; Yu et al. 2015). Thus, further research is needed to understand whether endorsing an evening chronotype has more pronounced negative impacts in older adults relative to middle‐aged adults.

The current study investigated how chronotype influenced sleep, alertness, and common, depression‐specific, and anxiety‐specific dimensions of internalising symptoms in an online sample (*N* = 652) of middle‐aged and older adults. Because eveningness often creates social jetlag with typical early morning work schedules, we predicted that eveningness, compared to morningness, would be associated with poorer sleep quality, lower alertness, and higher levels of internalising symptoms. For internalising symptoms, we explored whether eveningness would similarly or differentially influence common, depression‐specific and anxiety‐specific symptoms. Additionally, we explored how the effects of chronotype on sleep, internalising symptoms and alertness might unfold from middle age to late adulthood.

## 2 Materials and Methods

### 2.1 Participants

This study used data from a larger project investigating the effects of sleep and ageing on emotional memory consolidation (Denis et al. 2022; Niu, Utayde, Sanders, Cunningham, et al. 2024; Niu, Utayde, Sanders, Denis, et al. 2024). See Supporting Information Section VI for a detailed description of the larger project. A total of 1556 eligible participants were recruited through Prolific (https://www.prolific.co). Eligible participants needed to: (1) be at least 35 years old, (2) be fluent in English, (3) have normal or corrected‐to‐normal vision, (4) have no history of any diagnosed sleep, psychiatric, or neurological disorders, and (5) currently live in the United States. Of the 1556 eligible participants, 845 did not complete the entire study, and 59 were identified as outliers and removed from analyses (see Analyses for more details). The final sample included 652 participants (*M*_age_ = 53.66, SD_age_ = 11.46). Most participants self‐identified as white (83.4% White, 8.7% Black/African American, 6.1% Asian, 0.6% American Indian/Native Alaskan, 0.0% Native Hawaiian/Pacific Islander, and 1.1% other; 4.0% Hispanic/Latino/Spanish). Approximately half of the sample identified as female (55.7% female, 44.2% male, and 0.2% not reported). The median annual household income was 70,000 US dollars (range: 10,000 to 1,490,000). The Institutional Review Board at the University of Notre Dame approved the procedures. Participants provided written informed consent and were compensated for their participation.

### 2.2 Materials

#### 2.2.1 Chronotype

The Morningness‐Eveningness Questionnaire (MEQ) determines an individual's chronotype (Horne and Ostberg 1976) using both Likert‐scale (e.g., “During the first half‐hour after you wake up in the morning, how tired do you feel?”) and time‐scale questions (e.g., “At what time of night do you feel you become tired as a result of need for sleep?”). The global MEQ score ranges from 16 to 86, with higher scores indicative of a greater tendency towards morningness and lower scores indicative of a stronger preference for eveningness. Based on the global MEQ scores, participants were categorised into three chronotypes according to established criteria (Horne and Ostberg 1976). Amongst the 652 participants, 9.20% (*n* = 60) were evening chronotypes who preferred later sleep and rise times (MEQ range: 16–41, *M* = 36.32), 51.53% (*n* = 336) were intermediate chronotypes with neither morning nor evening preferences (MEQ range: 42–58, *M* = 50.98), and 39.26% (*n* = 256) were morning chronotypes who preferred earlier sleep onset and offset (MEQ range: 59–86, *M* = 65.94).

#### 2.2.2 Sleep Quality

The Pittsburgh Sleep Quality Index (PSQI) measures seven components of habitual sleep patterns over the past month: subjective sleep quality, sleep onset latency, sleep duration, sleep efficiency, sleep disturbances, sleep medication use, and daytime dysfunction (Buysse et al. 1989). Each component is scored from 0 to 3, resulting in a composite PSQI score ranging from 0 to 21, with higher scores indicating poorer sleep quality. We also scored four specific sleep metrics: (a) sleep onset latency (SOL: time taken to fall asleep after lights out), (b) total sleep time (TST: sleep duration during a planned sleep episode), and (c) sleep efficiency (SE: percentage of time spent asleep relative to total time in bed).

#### 2.2.3 Internalising Symptoms

The Mini‐Mood and Anxiety Symptom Questionnaire (Mini‐MASQ) measures 26 internalising symptoms of depression and anxiety (Casillas and Clark 2000). Participants rate how much they experienced each symptom over the past week (1 = not at all, 5 = extremely). The Mini‐MASQ measures three dimensions: (a) general distress, the shared component of depression and anxiety (e.g., “Felt uneasy”; eight items), (b) depression‐specific anhedonia (e.g., “Felt withdrawn from other people”; eight items), and (c) anxiety‐specific anxious arousal (e.g., “Hands were shaky”; 10 items).

#### 2.2.4 Behavioural Alertness

We used a 3‐min brief version of the Psychomotor Vigilance Test (PVT‐B) to assess behavioural alertness (Basner et al. 2011). The PVT‐B is more practical compared to the standard 10‐min PVT whilst still being sensitive to acute total sleep deprivation and chronic partial sleep loss (Basner et al. 2011). Participants were informed that they would be engaging in a reaction time task for 3 min. They were told to press the spacebar as quickly as possible whenever a red circle appeared on the screen, but to avoid reacting before the red circle appears. The interstimulus interval varies randomly from 1 to 4 s. Although participants did not undergo a practise session for the PVT, we calculated PVT scores using the median reaction time across all completed trials to minimise the impact of potential outliers on the data (Antler et al. 2022). The PVT‐B was programmed in jsPsych (de Leeuw 2015) and hosted online on Cognition.run (https://www.cognition.run).

#### 2.2.5 Subjective Alertness

The Stanford Sleepiness Scale (SSS) is a one‐item questionnaire that examines subjective alertness at specific moments in time (Shahid et al. 2012). Participants rate their current levels of alertness on a scale from 1 [feeling active, vital, alert, or wide awake] to 7 [no longer fighting sleep, sleep onset soon, having dream‐like thoughts].

### 2.3 Procedure

All participants completed an eligibility screening survey, providing information regarding their demographics, health, and sleep quality using the PSQI. Eligible participants were invited to two study sessions separated by 12 h, conducted between 7–11 AM and 7–11 PM. This timing was chosen to align with the larger project's investigation of sleep's impact on memory. The order of these sessions (evening‐morning vs. morning‐evening) was randomised. During both sessions, participants completed the SSS and PVT‐B as measures of subjective and behavioural alertness. In session one only, after the PVT‐B, participants filled out questionnaires to report on their chronotypes and internalising symptoms using the MEQ and Mini‐MASQ. All questionnaires were administered through Qualtrics (https://www.qualtrics.com). Data were collected between March 31st, 2023 and December 11th, 2023 in the United States.

### 2.4 Analyses

We tested the effects of chronotype and age on the following outcomes: (a) alertness: subjective and behavioural alertness both in the morning and evening, (b) sleep: overall sleep disturbance, sleep onset latency, total sleep time and sleep efficiency, and (c) internalising symptoms: common factor general distress, depression‐specific anhedonia and anxiety‐specific anxious arousal. Chronotype and age were analysed both as categorical and continuous predictors. For clarity, when chronotype was analysed as a categorical variable, it was categorised as evening, intermediate and morning chronotypes. When treated as a continuous variable, it was referred to as the morningness or eveningness scale. Of the 711 participants who completed the study, we excluded 59 for having outliers exceeding 3 standard deviations from the median. We performed list‐wise exclusions for the 59 participants because our analyses, that included all continuous predictors in a single model, required complete data for each participant. All data processing and analyses were carried out in R (R Core Team 2022). The threshold for statistical significance was set to *p* < 0.05, two‐tailed.

#### 2.4.1 Continuous Predictors

We conducted a multivariate regression analysis using the “lavaan” package in R (R Core Team 2022; Rosseel 2012). This analysis employed maximum likelihood estimation to examine how chronotype, age, and their interaction (all treated as continuous variables) simultaneously predicted 11 outcomes within a single model. Four of these were alertness outcomes (subjective and objective alertness both in the morning and evening and sleep), four were sleep outcomes (sleep disturbance, sleep onset latency, total sleep time and sleep efficiency), and three were internalising symptoms (general distress, anhedonia and anxious arousal). The two‐stage sharpened method was used to control the false discovery rate in the model (Benjamini et al. 2006). A post hoc sensitivity analysis using WebPower (Zhang and Yuan 2018) estimated that a minimum effect size of *β* = 0.06 would be detectable with 80% power in a sample size of 652 participants at α = 0.05.

#### 2.4.2 Categorical Predictors

To examine the main effects and interaction between chronotype and age for sleep and internalising symptoms, we conducted 3 (Chronotype Group: Evening, Intermediate, Morning) × 2 (Age Group: Middle Age [35–59], Older [60 and older]) between‐subjects Analyses of Variance (ANOVAs). Dependent variables included sleep disturbance, sleep onset latency, total sleep time, sleep efficiency, general distress, anhedonia and anxious arousal. The age cutoff for middle‐aged and older adults was chosen to be consistent with past projects in our research group for cross‐experiment comparisons (Denis et al. 2022; Niu, Utayde, Sanders, Cunningham, et al. 2024; Niu, Utayde, Sanders, Denis, et al. 2024). To test if chronotype or age further interacts with time of day, we conducted 3 (Chronotype Group: Evening, Intermediate, Morning) × 2 (Age Group: Middle Age [35–59], Older [60 and older]) × 2 (Time of Day: 7–11 AM, 7–11 PM) mixed‐effects ANOVAs for both behavioural and subjective alertness.

For all ANOVA models, effect sizes were estimated using partial omega‐squared (*ω* ^*2*^ *p*), an unbiased measure of population effects that is less sensitive to unequal sample sizes across groups (Olejnik and Algina 2003). A post hoc sensitivity analysis using G*Power (Faul et al. 2007) indicated that, with a sample size of 652 participants, the minimum detectable effect size for achieving 80% power at *α* = 0.05 was *ω* ^*2*^ *p* = 0.01 (or Cohen's *f* = 0.12). Significant main effects and interactions were further examined with pairwise *t*‐tests, applying Welch's approximation for unequal variances in independent samples *t*‐tests (Ruxton 2006). Effect sizes for these pairwise comparisons were calculated using Cohen's *d*, and *p*‐values were adjusted using the Bonferroni correction.

## 3 Results

Eveningness was significantly correlated with a later midpoint of sleep (i.e., the half point between sleep onset and offset), *r* = 0.66, *p* < 0.001. When binned into groups, compared to morning chronotypes (*M* = 02:07:07, SD = 00:54:18), both evening (*M* = 04:46:22, SD = 01:21:30, *t*(72) =14.40, *p* < 0.001, *d* = 2.30) and intermediate chronotypes (*M* = 03:13:46, SD = 01:04:59, *t*(585) = 13.58, *p* < 0.001, *d* = 1.11) had significantly later midpoints of sleep. Evening chronotypes also had a later midpoint of sleep than intermediate chronotypes (*t*(73) = 8.34, *p* < 0.001, *d* = 1.26).

See Table 1 for demographic characteristics grouped by chronotype and age. Increasing age was significantly correlated with a greater tendency towards morningness (*r* = 0.20, *p* < 0.001). Compared to morning chronotypes (*M* = 56.29, SD = 10.97), both evening (*M* = 48.77, SD = 11.82, *t*(84) = −4.49, *p* < 0.001, *d* = −0.66) and intermediate chronotypes (*M* = 52.53, SD = 11.33, *t*(558) = −4.07, *p* < 0.001, *d* = 0.34) were significantly younger. There were no significant differences in eveningness‐morningness tendencies between females and males (*F*(1, 647) = 0.19, *p* = 0.663, *ω* ^*2*^ *p* < 0.01), nor was there a significant interaction with age (*F*(1, 647) = 2.35, *p* = 0.126, *ω* ^*2*^ *p* < 0.01).

**TABLE 1:** Demographic characteristics for MEQ subtypes and age groups.

	Middle‐aged (35–59)			Older (60 and above)		
	Evening	Intermediate	Morning	Evening	Intermediate	Morning
Sample size	*n* = 48	*n* = 230	*n* = 140	*n* = 12	*n* = 106	*n* = 116
Age (years)	44.17 (7.87)	46.32 (7.19)	48.13 (6.83)	67.17 (4.75)	66.00 (5.39)	66.13 (5.63)
PSQI medication	0.15 (0.55)	0.19 (0.57)	0.34 (0.85)	0.50 (1.17)	0.58 (1.10)	0.34 (0.91)
Sex: *n* (%)						
Female	20 (41.67%)	116 (50.43%)	72 (51.43%)	11 (91.67%)	72 (67.92%)	72 (62.07%)
Male	28 (58.33%)	114 (49.57%)	68 (48.57%)	1 (8.33%)	34 (32.08%)	43 (37.07%)
Not reported	0 (0.00%)	0 (0.00%)	0 (0.00%)	0 (0.00%)	0 (0.00%)	1 (0.86%)
Ethnicity: *n* (%)						
Hispanic, Latino, or Spanish	7 (14.58%)	10 (4.35%)	4 (2.86%)	0 (0.00%)	5 (4.72%)	0 (0.00%)
None	41 (85.42%)	220 (95.65%)	136 (97.14%)	12 (100.00%)	101 (95.28%)	116 (100.00%)
Race: *n* (%)						
White	34 (70.83%)	183 (79.57%)	121 (86.43%)	12 (100.00%)	93 (87.74%)	101 (87.07%)
Black or African American	5 (10.42%)	23 (10.00%)	10 (7.14%)	0 (0.00%)	7 (6.60%)	12 (10.34%)
Asian	7 (14.58%)	22 (9.57%)	6 (4.29%)	0 (0.00%)	3 (2.83%)	2 (1.72%)
American Indian or Alaska native	0 (0.00%)	1 (0.43%)	1 (0.71%)	0 (0.00%)	2 (1.89%)	0 (0.00%)
Other	2 (4.17%)	1 (0.43%)	2 (1.43%)	0 (0.00%)	1 (0.94%)	1 (0.86%)
Annual income: M (SD)	67,732 (62358)	90,632 (69160)	99,099 (63590)	48,255 (31,486)	81,064 (59,206)	86,842 (141949)

*Note*: Means (standard deviations) are displayed for age, MEQ, and annual income. Sample size (percentages) are displayed for biological sex, ethnicity and race. MEQ, the Morningness‐Eveningness Questionnaire. PSQI Medication, a component of the Pittsburgh Sleep Quality Index, which asks, “During the past month, how often have you taken medicine to help you sleep (prescribed or over the counter)?”, where 0 indicates “Not during the past month” and 1 indicates “Less than once a week”.

Supporting Informations Table S1–S6 summarise descriptive statistics and bivariate correlations for all key study variables. Tables S7–S11 present the results of ANOVAs and pairwise *t*‐tests for the main and interactive effects of chronotype and age, both as categorical predictors. Tables S12–S14 detail full model results of multivariate regressions that treated chronotype and age as continuous variables.

### 3.1 Alertness

#### 3.1.1 Effect of Chronotype

**FIGURE 1:**
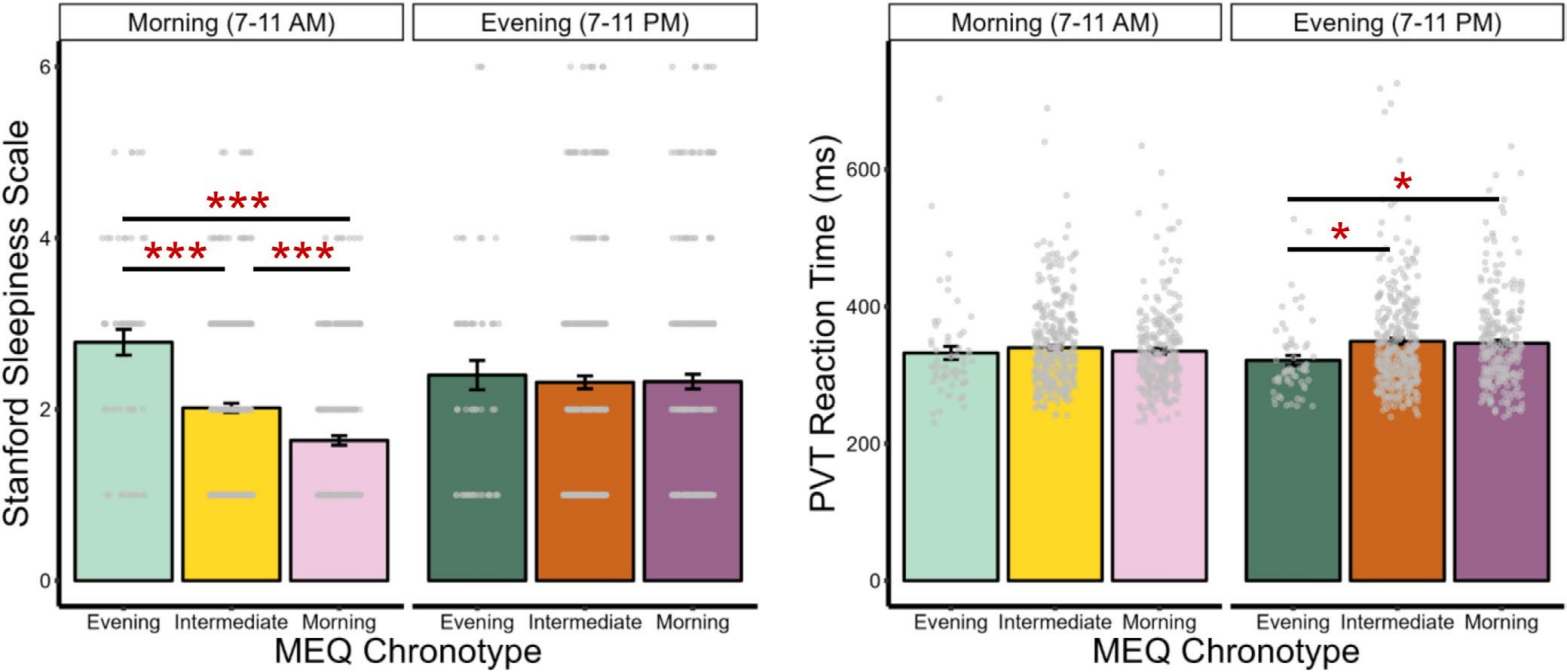
The interactive effects of chronotype and time of day on subjective and behavioural alertness. *Note*: MEQ, the Morningness‐Eveningness Questionnaire, where evening chronotypes prefer later sleep and rise times, intermediate chronotypes have neither morning nor evening preferences, and morning chronotypes prefer earlier sleep onset and offset. Sandford Sleepiness Scale, where higher scores indicate feeling sleepier, or lower subjective alertness. PVT, the Psychomotor Vigilance Task, where faster median reaction times indicate greater behavioural alertness. Significance levels for Bonferroni‐corrected *p*‐values are denoted as follows: *< 0.050, ***< 0.001. Error bars represent ±1 standard error.

When examining the effects of chronotype group on subjective alertness separately in the morning (7–11 AM) and evening (7–11 PM), a significant chronotype‐by‐time‐of‐day interaction emerged (*F*(2, 646) = 17.67, *p* < 0.001, *ω* ^*2*^ *p* = 0.05; Figure 1). Post hoc pairwise comparisons showed that, only in the AM, evening chronotypes reported feeling significantly sleepier (or lower subjective alertness) compared to both intermediate (*t*(75) = 4.78, *p* < 0.001, *d* = 0.70) and morning chronotypes (*t*(76) = 7.15, *p* < 0.001, *d* = 1.11). Intermediate chronotypes also reported lower subjective alertness compared to morning chronotypes (*t*(578) = 4.90, *p* < 0.001, *d* = 0.40). However, these comparisons were not observed in the PM (*ps* > 0.9; Table S10). Consistent with this interaction, eveningness (as a continuous variable) was linked to lower subjective alertness in the AM (*β* = 0.59, *p* = 0.007) but not PM (*β* = 0.18, *p* = 0.343).

A significant chronotype‐by‐time‐of‐day interaction also emerged for behavioural alertness (*F*(2, 646) = 4.20, *p* = 0.015, *ω* ^*2*^ *p* = 0.01; Figure 1). That is, when the PVT was administered in the PM, evening chronotypes demonstrated significantly faster reaction times (indicating higher behavioural alertness), compared to both intermediate (*t*(111) = −3.26, *p* = 0.012, *d* = −0.39) and morning chronotypes (*t*(106) = −2.96, *p* = 0.048, *d* = −0.40). However, eveningness (as a continuous variable) was not correspondingly associated with increased behavioural alertness in the PM (*β* = 0.02, *p* = 0.814). No significant differences were observed between chronotype groups in the AM assessment either (*ps* > 0.9; Table S10).

#### 3.1.2 Effect of Time of Day

When examining the effect of time of day on alertness within each chronotype, both intermediate (*t*(335) = −3.80, *p* < 0.001, *d* = −0.21) and morning chronotypes (*t*(255) = −7.90, *p* < 0.001, *d* = −0.49) reported feeling more alert in the AM than PM. Morning chronotypes also exhibited significantly higher behavioural alertness in the AM compared to their own performance in the PM (*t*(255) = −2.89, *p* = 0.024, *d* = −0.18). No other differences were found between AM and PM assessments (*ps* > 0.9; Table S10).

#### 3.1.3 Age Interactions

This chronotype‐by‐time‐of‐day interaction in alertness differed significantly between middle‐aged and older adults for subjective (*F*(2, 646) = 3.11, *p* = 0.045, *ω* ^*2*^ *p* = 0.01), but not for behavioural alertness (*F*(2, 646) = 1.64, *p* = 0.195, *ω* ^*2*^ *p* < 0.01). Specifically, older evening chronotypes (*t*(11) = −3.77, *p = 0*.018, *d* = −1.09), but not middle‐aged ones (*t*(47) = −0.88, *p =* 1.00, *d* = −0.13), reported feeling less sleepy (i.e., more alert) in the PM relative to their own subjective alertness in the AM. In line with this, older age, on a continuous scale, was associated with feeling more alert in the evening (*β* = −0.28, *p* = 0.045), but not in the morning (*β* = −0.15, *p* = 0.186).

### 3.2 Sleep

The main effect of chronotype group was significant for overall sleep disturbance (*F*(2, 646) = 7.56, *p* = 0.001, *ω* ^*2*^ *p* = 0.02) and sleep onset latency (*F*(2, 646) = 6.82, *p* = 0.001, *ω* ^*2*^ *p* = 0.02). However, no other significant main effects were observed, nor did any effects interact with age, whether both variables were analysed categorically or continuously (*ps* > 0.3; Tables S7 and S12). Post hoc pairwise comparisons revealed that evening chronotypes reported significantly higher levels of sleep disturbance than morning chronotypes (*t*(87) = 3.44, *p* = 0.024, *d* = 0.50). In line with these results, treating chronotype as a continuous predictor revealed that eveningness was significantly associated with greater sleep disturbance (*β* = 0.56, *p* = 0.015) as well as longer sleep onset latency (*β* = 0.46, *p* = 0.043). No other significant pairwise comparisons were observed (*ps* > 0.9; Table S8).

**FIGURE 2:**
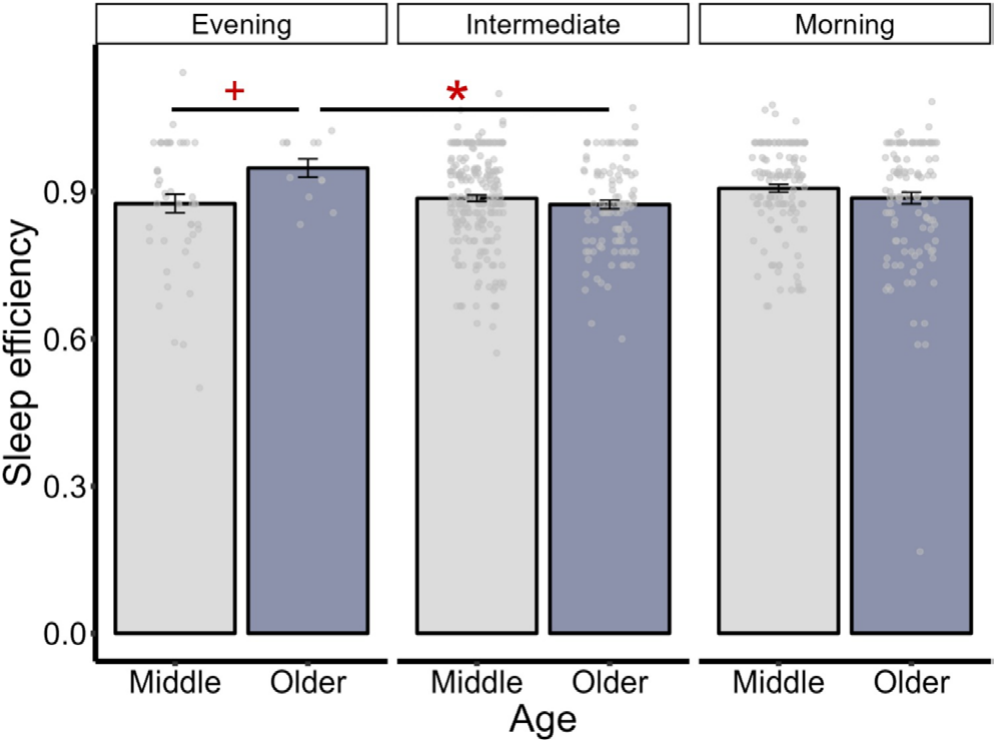
Pairwise comparisons for the effects of age on sleep efficiency within each chronotype. *Note*: Middle = aged between 35 and 59. Older = aged 60 or above. Sleep efficiency, percentage of time spent asleep relative to total time in bed. Significance levels for Bonferroni‐corrected *p*‐values are denoted as follows: ^+^, < 0.010, *< 0.050. Error bars represent ±1 standard error.

We found a significant chronotype by age interaction for sleep efficiency (*F*(2, 646) = 3.28, *p* = 0.038, *ω* ^*2*^ *p* = 0.01). Compared to older adults who were classified as evening chronotypes, older adults with intermediate chronotypes (*t*(17) = −3.59, *p* = 0.024, *d* = −0.93) and middle‐aged adults with evening chronotypes (*t*(36) = −2.73, *p* = 0.060, *d* = −0.70) had lower sleep efficiency, although the later comparison was not significant (Figure 2).

### 3.3 Internalising Symptoms

**FIGURE 3:**
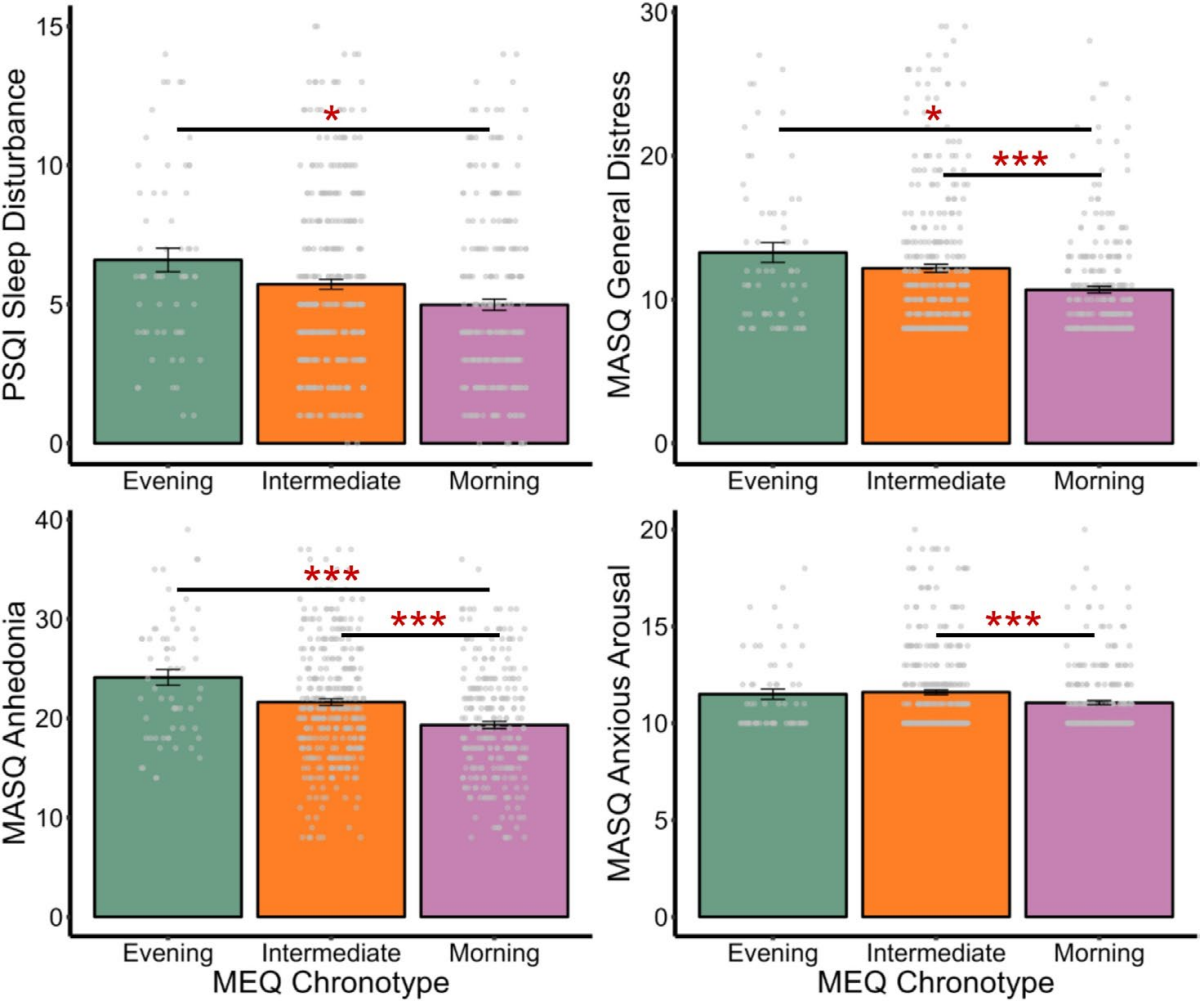
Pairwise comparisons for the effects of chronotypes on sleep and internalising symptoms. *Note*: MASQ, the Mood and Anxiety Symptom Questionnaire, measures (a) general distress, a shared symptom between depression and anxiety, (b) anhedonia, a depression‐specific symptom, and (c) anxious arousal, an anxiety‐specific symptom. MEQ, the Morningness‐Eveningness Questionnaire, where evening chronotypes prefer later sleep and rise times, intermediate chronotypes have neither morning nor evening preferences, and morning chronotypes prefer earlier sleep onset and offset. PSQI, the Pittsburgh Sleep Quality Index, where higher composite scores represent greater sleep disturbance. Significance levels for Bonferroni‐corrected *p*‐values are denoted as follows: *, < 0.050, ***< 0.001. Error bars represent ±1 standard error.

The main effect of chronotype group was significant for general distress (*F*(2, 646) = 11.71, *p* < 0.001, *ω* ^*2*^ *p* = 0.03), anhedonia (*F*(2, 646) = 20.09, *p* < 0.001, *ω* ^*2*^ *p* = 0.06), and anxious arousal (*F*(2, 646) = 5.10, *p* = 0.006, *ω* ^*2*^ *p* = 0.01). However, chronotype and age, either when binned into groups or treated as continuous variables, did not interact with each other to predict any of the internalising symptoms (*ps* > 0.7; Tables S7 and S12). Compared to evening chronotypes, morning chronotypes reported significantly lower levels of general distress (*t*(73) = −3.55, *p* = 0.024, *d* = −0.56) and anhedonia (*t*(85) = −5.47, *p* < 0.001, *d* = −0.80) (Figure 3). Similarly, morning chronotypes showed less severe general distress (*t*(589) = −4.14, *p* < 0.001, *d* = −0.34), anhedonia (*t*(560) = −4.67, *p* < 0.001, *d* = −0.39), and anxious arousal (*t*(590) = −3.27, *p* = 0.024, *d* = −0.27), when compared to intermediate chronotypes (Figure 1). In comparison, eveningness, on a continuous scale, was associated with more severe symptoms of anhedonia (*β* = 0.53, *p* = 0.015), but not associated with general distress (*β* = 0.26, *p* = 0.181) or anxious arousal (*β* = 0.26, *p* = 0.186).

## 4 Discussion

Our study examined the link between chronotype and well‐being in middle and late adulthood. Results demonstrated that middle‐aged and older adults who endorsed evening chronotypes were more likely to experience worse sleep quality, report higher levels of common, depression‐specific and anxiety‐specific internalising symptoms, and feel less alert in the morning. Evening chronotypes also demonstrated enhanced behavioural alertness when they were tested later rather than earlier in the day, suggesting a potential advantage during their optimal performance period. However, these effects did not further interact with age.

We replicated existing research demonstrating that evening chronotypes tend to have worse sleep quality in late adulthood (Suh et al. 2017), and further demonstrated that this association extended to middle age. As we age, we experience increased difficulties regulating our sleep–wake cycles in response to social jet lag (Didikoglu et al. 2019; Hood and Amir 2017; Kim et al. 2022). This is potentially due to the age‐related reduction of the endocrine and neuroendocrine rhythms, including core body temperature, cortisol, and melatonin release (Didikoglu et al. 2019; Hood and Amir 2017; Kim et al. 2022). In addition, unlike younger adults who can compensate for sleep loss with increased slow‐wave sleep amount and intensity to achieve physical and cognitive restoration, middle‐aged and older adults exhibit reduced homeostatic control to upregulate slow‐wave activity as sleep‐dependent recovery from circadian misalignment (Lazar et al. 2015). Sleep disturbances stemming from having an evening chronotype may, in turn, perpetuate mood and anxiety disorders through alterations in the sensitivity of serotonin, dopamine, and noradrenaline neurotransmitter receptors (Monti 2011). Relatedly, evening chronotypes may be more prone to accelerated cognitive ageing due to chronic sleep disturbance and adverse mental health outcomes (Hicks et al. 2023; Leahy et al. 2024; Suh et al. 2017).

Whilst eveningness was associated with greater sleep disturbance in our study, a surprising finding emerged: older adults classified as evening chronotypes reported higher sleep efficiency compared to their intermediate chronotype counterparts. This finding also contradicts previous research that typically demonstrates decreased sleep efficiency in evening chronotypes (Didikoglu et al. 2019; Sinha et al. 2023). Several factors may contribute to this unexpected finding. First, although our study excluded individuals with diagnosed sleep disorders, our data show that older evening and intermediate chronotypes (Table 1) were often taking sleep‐aid medications to improve sleep efficiency. Future studies should explicitly examine the potential confounding influence of sleep‐aid medications to better understand the complex interplay between chronotype, ageing and sleep efficiency. Next, there were 12 participants in our study who were classified as older evening chronotypes, representing 1.84% of the total sample. This is consistent with the percentage of older evening chronotypes revealed in prior research (Yoon et al. 1999) and is likely driven by age‐related changes, such as earlier onset of melatonin production and heightened sensitivity to light exposure (Adan et al. 2012; Hood and Amir 2017). Although our statistical analyses accounted for unequal sample sizes across groups, future research should over‐recruit from this chronotype and age group to test the generalisability of this effect (Olejnik and Algina 2003; Ruxton 2006).

In addition to sleep disturbance, eveningness likely contributes to more severe internalising symptoms through reduced daily light exposure (Böhmer et al. 2021; Roenneberg et al. 2007), and the chronic stress of constantly adapting to morning schedules that are desynchronized with their internal diurnal preferences (Roenneberg et al. 2019; Zou et al. 2022). Building upon prior work on the association between evening chronotype and both depression (Norbury 2021; Zou et al. 2022) and anxiety symptoms (Antypa et al. 2016; Cox and Olatunji 2016), this study further explored the influence of chronotype on specific dimensions of internalising psychopathology. Our findings reveal that evening chronotype is a transdiagnostic risk factor for general distress, the shared symptom between depression and anxiety, as well as depression‐specific anhedonia and anxiety‐specific anxious arousal. Notably, the association was the strongest for anhedonia, perhaps in part because evening chronotypes tend to experience reduced sunlight exposure, which may disrupt dopamine‐mediated reward processing and contribute to loss of pleasure or interest (Böhmer et al. 2021; Dresp‐Langley 2023). This suggests that the previously observed link between eveningness and individual diagnoses of depression and anxiety might be partially explained by the extensive comorbidity between depression and anxiety symptoms (Hankin et al. 2016). These results highlight the importance of targeting chronotype in the assessment and treatment for individuals struggling with internalising disorders (Zou et al. 2022).

We hypothesised that older adults would experience more pronounced negative consequences from evening chronotype than middle‐aged adults, due to their reduced endogenous control over circadian rhythms (Lazar et al. 2015). However, we found no significant age‐related differences in the association between eveningness and internalising symptoms. One possible explanation is that healthy ageing is linked to a greater sense of meaning in life and improved psychological well‐being, despite physical and cognitive decline (Höglund et al. 2020). This “positivity effect” in older adults, coupled with their increased flexibility in daily schedules due to retirement, may buffer against the negative impact of eveningness (Barber and Kim 2021). Our bivariate correlations, which showed older age associated with less severe general distress, anhedonia, and anxious arousal, support this interpretation (Table S3).

Extending previous work that has observed a dissociation between perceived and objective measures of alertness (Hao et al. 2021), our findings demonstrated that evening chronotype is associated with lower subjective alertness in the AM and higher behavioural alertness in the PM. However, we did not find evidence for the reverse pattern: eveningness was not associated with higher subjective alertness in the PM or lower behavioural alertness in the AM. This dissociation may be partially explained by diurnal cortisol rhythm. Specifically, due to the cortisol awakening response, individuals across all chronotypes experience a surge in cortisol levels upon waking up, which potentially leads to equivalent behavioural alertness in early morning hours (O'Byrne et al. 2021). However, evening chronotypes may experience a blunted cortisol awakening response due to chronic sleep disturbances from a misalignment between their sleep–wake cycles and external demands (Bailey and Heitkemper 2001; Petrowski et al. 2020). As subjective alertness is more sensitive to poor sleep quality compared to psychomotor speed, it may better capture this subtle decline in early morning arousal levels (Antler et al. 2022; St. Hilaire et al. 2017).

In contrast, as the day progresses, all individuals may experience a general decline in subjective alertness due to the accumulation of fatigue from societal commitments (O'Byrne et al. 2021). However, retired older adults, who are less constrained by these demands, may experience a less pronounced decline in subjective alertness throughout the day. This may partly explain why older evening chronotypes in our study reported feeling more alert in the PM compared to the AM. Relatedly, evening chronotypes may exhibit enhanced behavioural alertness later in the day due to their delayed peak in cortisol levels (Bailey and Heitkemper 2001; Petrowski et al. 2020). Therefore, although it may be challenging for evening chronotypes to adjust to societal schedules that favour morning activities, they can optimise their performance by engaging in demanding tasks during their peak alertness periods.

## 5 Limitations and Future Directions

Whilst the current online study recruited participants from various US communities, the predominately White participant pool limits the generalizability of our findings to racial/ethnic minorities. Second, our findings may not generalise to the broader ageing population with limited technological access necessary to participate in an online study. Next, the current study assessed sleep quality using questionnaire measures, but it is important to acknowledge that middle‐aged and older adults may have difficulty accurately recalling their sleep experiences (Miner et al. 2022; Zak et al. 2022). The online design precluded the collection of overnight sleep data, preventing us from determining how objectively monitored sleep architecture aligns with self‐report sleep quality. Future studies would benefit from incorporating in‐lab assessments of objective sleep measures in representative samples across various racial/ethnic backgrounds and health conditions. Prior research indicates that females are more likely to be morning chronotypes than males, although this sex difference diminishes after menopause (Adan et al. 2012; Hood and Amir 2017). Future studies should investigate the intricate interplay between age and sex on chronotype and related implications for health and performance outcomes.

In addition, the study collected data between March and December and did not control for seasonal influences that could affect the phase of the circadian rhythmicity—a key component of chronotype (Díaz‐Morales et al. 2017). For example, reduced daylight in winter is linked to increased eveningness and depressive symptoms (Murray et al. 2003; Natale et al. 2005). Relatedly, another limitation of the study is the variations in session timing within the four‐hour testing windows (7–11 AM or 7–11 PM), which might capture different points along the diurnal fluctuations of mood and alertness (O'Byrne et al. 2021). Future research should account for individuals' susceptibility to seasonal and time‐of‐day influences for a more comprehensive understanding of chronotype.

## 6 Conclusions

Endorsing an evening chronotype was associated with greater sleep disturbance, morning sleepiness and internalising symptoms. Notably, the negative impacts of eveningness were not more pronounced in older adults compared to middle‐aged adults, challenging the notion of increased vulnerability with age, possibly because prior research primarily compared older and younger adults. Additionally, the study provides initial evidence that eveningness was more strongly associated with depression‐specific symptoms, rather than anxiety‐specific or shared ones. However, evening chronotypes outperformed morning and intermediate chronotypes on a behavioural alertness test administered later in the day. Overall, these results highlight the importance of considering chronotype and circadian misalignment in mental and physical health assessment, as well as their potential relevance for promoting healthy ageing.

## Author Contributions

**Xinran Niu:** conceptualization, writing – original draft, methodology, software, formal analysis, project administration, data curation, visualization, validation, investigation. **Kristin E. G. Sanders:** formal analysis, supervision, writing – review and editing, software. **Elizabeth A. Kensinger:** funding acquisition, writing – review and editing, supervision, resources. **Jessica D. Payne:** supervision, writing – review and editing, funding acquisition, resources.

## Conflicts of Interest

The authors declare no conflicts of interest.

## Supporting information


**Data S1.** Supporting Information.

## Data Availability

The data that support the findings of this study are openly available in OSF at https://doi.org/10.17605/OSF.IO/2WV35.
