# Supplementary material for: Eveningness in Middle‐Aged and Older Adults: Associations With Sleep, Internalising Symptoms, and Alertness
Source: J Sleep Res. 2025 Jun 2;35(1):e70104. doi: 10.1111/jsr.70104 (PMC12856121; doi:10.1111/jsr.70104)
Supplement: Supplementary file 1 — Data S1. Supporting Information. [file JSR-35-e70104-s001.docx]

**Contents**

[I. Descriptive Statistics 1](#_Toc196259245)

[II. Bivariate Correlations 1](#_Toc196259246)

[III. Sleep and Internalizing Symptoms 5](#_Toc196259247)

[IV. Behavioral and Subjective Alertness 7](#_Toc196259248)

[V. Multivariate Regression Models 10](#_Toc196259249)

[VI. Description of the Larger Projects 14](#_Toc196259250)

[VII. Measurement Invariance Testing 15](#_Toc196259251)

[VIII. Comparing Extreme Values 20](#_Toc196259252)

[IX. Analyses Including Outliers 23](#_Toc196259253)

[X. References 25](#_Toc196259254)

# I. Descriptive Statistics

**Table S1**

*Means (Standard Deviations) for Key Study Variables in Total Sample and MEQ Subtypes in Middle-Aged Adults (Aged 35-59)*

|  |  | **Middle-Aged (35-59)** | | |
| --- | --- | --- | --- | --- |
|  | **Total Sample** | ***Evening*** | ***Intermediate*** | ***Morning*** |
| MEQ-Composite | 55.50 (10.53) | 36.31 (4.16) | 51.12 (4.55) | 65.19 (4.90) |
| MPS (hh:mm:ss) | 02:56:07 (01:18:27) | 04:45:53 (01:25:03) | 03:10:46 (01:05:39) | 02:09:51 (00:51:55) |
| Age | 53.66 (11.46) | 44.17 (7.87) | 46.32 (7.19) | 48.13 (6.83) |
| Morning PVT (ms) | 337.23 (69.90) | 322.09 (48.79) | 338.13 (66.41) | 334.31 (77.91) |
| Evening PVT (ms) | 345.64 (76.45) | 320.22 (57.44) | 348.29 (88.14) | 346.26 (74.34) |
| Morning SSS | 1.94 (1.03) | 2.85 (1.22) | 2.01 (1.00) | 1.76 (0.95) |
| Evening SSS | 2.33 (1.36) | 2.65 (1.31) | 2.43 (1.38) | 2.39 (1.36) |
| PSQI-TST (min) | 414.50 (64.04) | 400.00 (74.61) | 406.94 (62.49) | 420.21 (50.35) |
| PSQI-SOL (min) | 21.15 (17.84) | 28.31 (25.48) | 22.63 (19.27) | 17.66 (13.60) |
| PSQI-SE (%) | 0.89 (0.11) | 0.88 (0.13) | 0.89 (0.10) | 0.91 (0.09) |
| PSQI-Composite | 5.51 (3.25) | 6.90 (3.29) | 5.70 (3.19) | 4.79 (2.97) |
| General Distress | 11.69 (4.69) | 13.75 (5.30) | 12.65 (5.45) | 11.21 (4.24) |
| Anhedonia | 20.95 (6.17) | 24.69 (6.12) | 21.99 (6.22) | 19.91 (5.78) |
| Anxious Arousal | 11.38 (2.10) | 11.29 (1.68) | 11.59 (2.36) | 11.22 (1.94) |
| *Note.* MEQ, the Morningness-Eveningness Questionnaire, where higher composite scores indicate preferences for morningness. PSQI, the Pittsburgh Sleep Quality Index, where higher composite scores represent greater sleep disturbance. PVT, the Psychomotor Vigilance Test, where slower reaction times correspond to lower behavioral alertness. SSS, the Stanford Sleepiness Scale, where higher ratings indicate lower subjective alertness. TST, total sleep time. SOL, sleep onset latency. SE, sleep efficiency. MPS, midpoint of sleep. | | | | |

**Table S2**

*Means (Standard Deviations) for Key Study Variables in Total Sample and MEQ Subtypes in Older Adults (Aged* ≥ 60 Years)

|  |  | **Older (60 and above)** | | |
| --- | --- | --- | --- | --- |
|  | **Total Sample** | ***Evening*** | ***Intermediate*** | ***Morning*** |
| MEQ-Composite | 55.50 (10.53) | 36.33 (5.84) | 50.67 (5.10) | 66.84 (5.25) |
| MPS (hh:mm:ss) | 02:56:07 (01:18:27) | 04:48:20 (01:08:40) | 03:20:15 (01:03:21) | 02:03:48 (00:57:05) |
| Age | 53.66 (11.46) | 67.17 (4.75) | 66.00 (5.39) | 66.13 (5.63) |
| Morning PVT (ms) | 337.23 (69.90) | 373.12 (132.25) | 343.75 (72.09) | 335.53 (62.16) |
| Evening PVT (ms) | 345.64 (76.45) | 326.05 (53.77) | 351.56 (72.25) | 346.78 (64.74) |
| Morning SSS | 1.94 (1.03) | 2.50 (0.90) | 2.03 (1.03) | 1.49 (0.77) |
| Evening SSS | 2.33 (1.36) | 1.42 (0.79) | 2.08 (1.31) | 2.24 (1.37) |
| PSQI-TST (min) | 414.50 (64.04) | 415.00 (63.75) | 425.94 (68.71) | 418.06 (70.87) |
| PSQI-SOL (min) | 21.15 (17.84) | 22.92 (21.16) | 20.85 (16.11) | 19.56 (15.82) |
| PSQI-SE (%) | 0.89 (0.11) | 0.95 (0.06) | 0.87 (0.09) | 0.89 (0.13) |
| PSQI-Composite | 5.51 (3.25) | 5.42 (3.18) | 5.75 (3.33) | 5.22 (3.43) |
| General Distress | 11.69 (4.69) | 11.33 (5.35) | 11.14 (3.92) | 10.04 (2.91) |
| Anhedonia | 20.95 (6.17) | 21.83 (6.13) | 20.83 (5.71) | 18.60 (5.85) |
| Anxious Arousal | 11.38 (2.10) | 12.33 (3.08) | 11.64 (2.25) | 10.86 (1.44) |
| *Note.* MEQ, the Morningness-Eveningness Questionnaire, where higher composite scores indicate preferences for morningness. PSQI, the Pittsburgh Sleep Quality Index, where higher composite scores represent greater sleep disturbance. PVT, the Psychomotor Vigilance Test, where slower reaction times correspond to lower behavioral alertness. SSS, the Stanford Sleepiness Scale, where higher ratings indicate lower subjective alertness. TST, total sleep time. SOL, sleep onset latency. SE, sleep efficiency. MPS, midpoint of sleep. | | | | |

# II. Bivariate Correlations

**Table S3**

*Bivariate Correlations for Key Study Variables in the Total Sample*

|  | 1 | 2 | 3 | 4 | 5 | 6 | 7 | 8 | 9 | 10 | 11 | 12 | 13 | 14 |  |
| --- | --- | --- | --- | --- | --- | --- | --- | --- | --- | --- | --- | --- | --- | --- | --- |
| 1. MEQ-Composite | 1 |  |  |  |  |  |  |  |  |  |  |  |  |  |  |
| 2. MPS | -0.66^***^ | 1 |  |  |  |  |  |  |  |  |  |  |  |  |  |
| 3. Age | 0.20^***^ | -0.08^*^ | 1 |  |  |  |  |  |  |  |  |  |  |  |  |
| 4. Morning PVT | -0.02 | -0.02 | 0.06 | 1 |  |  |  |  |  |  |  |  |  |  |  |
| 5. Evening PVT | 0.06 | -0.07 | 0.00 | 0.51^***^ | 1 |  |  |  |  |  |  |  |  |  |  |
| 6. Morning SSS | -0.37^***^ | 0.23^***^ | -0.09^*^ | 0.04 | 0.00 | 1 |  |  |  |  |  |  |  |  |  |
| 7. Evening SSS | 0.01 | -0.07 | -0.14^***^ | -0.02 | 0.14^***^ | 0.27^***^ | 1 |  |  |  |  |  |  |  |  |
| 8. TST | 0.10^**^ | 0.02 | 0.07 | 0.01 | -0.01 | -0.09^*^ | -0.08^*^ | 1 |  |  |  |  |  |  |  |
| 9. SOL | -0.16^***^ | 0.05 | -0.05 | 0.08^*^ | 0.03 | 0.23^***^ | 0.09^*^ | -0.26^***^ | 1 |  |  |  |  |  |  |
| 10. SE | 0.09^*^ | 0.00 | -0.04 | -0.02 | -0.01 | -0.25^***^ | -0.20^***^ | 0.50^***^ | -0.44^***^ | 1 |  |  |  |  |  |
| 11. Sleep Disturbance | -0.18^***^ | 0.06 | 0.00 | 0.03 | 0.00 | 0.30^***^ | 0.25^***^ | -0.64^***^ | 0.58^***^ | -0.68^***^ | 1 |  |  |  |  |
| 12. General Distress | -0.19^***^ | 0.11^**^ | -0.17^***^ | 0.01 | -0.03 | 0.28^***^ | 0.30^***^ | -0.17^***^ | 0.14^***^ | -0.20^***^ | 0.35^***^ | 1 |  |  |  |
| 13. Anhedonia | -0.25^***^ | 0.07 | -0.18^***^ | 0.09^*^ | 0.00 | 0.31^***^ | 0.27^***^ | -0.18^***^ | 0.15^***^ | -0.19^***^ | 0.34^***^ | 0.56^***^ | 1 |  |  |
| 14. Anxious Arousal | -0.11^**^ | 0.08^*^ | -0.02 | -0.02 | -0.02 | 0.22^***^ | 0.17^***^ | -0.18^***^ | 0.18^***^ | -0.27^***^ | 0.35^***^ | 0.39^***^ | 0.22^***^ | 1 |  |
| *Note.* MEQ, the Morningness-Eveningness Questionnaire, where higher composite scores indicate preferences for morningness. MPS, midpoint of sleep. PVT, the Psychomotor Vigilance Test, where slower reaction times correspond to lower behavioral alertness. SSS, the Stanford Sleepiness Scale, where higher ratings indicate lower subjective alertness. TST, total sleep time. SOL, sleep onset latency. SE, sleep efficiency. Significant effects after the two-stage controlling procedure for the false discovery rate are in bold: ^*^, <.05, ^**^, <.01, ^***^ <.001. | | | | | | | | | | | | | | | |

**Table S4**

*Bivariate Correlations for Key Study Variables in Evening Chronotypes*

|  | 1 | 2 | 3 | 4 | 5 | 6 | 7 | 8 | 9 | 10 | 11 | 12 | 13 | 14 |
| --- | --- | --- | --- | --- | --- | --- | --- | --- | --- | --- | --- | --- | --- | --- |
| 1. MEQ-Composite | 1 |  |  |  |  |  |  |  |  |  |  |  |  |  |
| 2. MPS | -0.17 | 1 |  |  |  |  |  |  |  |  |  |  |  |  |
| 3. Age | 0.11 | -0.06 | 1 |  |  |  |  |  |  |  |  |  |  |  |
| 4. Morning PVT | 0.03 | -0.09 | 0.14 | 1 |  |  |  |  |  |  |  |  |  |  |
| 5. Evening PVT | -0.13 | 0.06 | -0.04 | 0.47^***^ | 1 |  |  |  |  |  |  |  |  |  |
| 6. Morning SSS | -0.32^*^ | 0.05 | -0.18 | 0.10 | -0.05 | 1 |  |  |  |  |  |  |  |  |
| 7. Evening SSS | -0.10 | 0.19 | -0.32^*^ | 0.04 | 0.30^*^ | 0.21 | 1 |  |  |  |  |  |  |  |
| 8. TST | 0.23 | 0.16 | 0.13 | 0.06 | -0.03 | -0.18 | 0.00 | 1 |  |  |  |  |  |  |
| 9. SOL | -0.08 | 0.04 | -0.09 | -0.15 | -0.21 | 0.27^*^ | -0.04 | -0.38^**^ | 1 |  |  |  |  |  |
| 10. SE | 0.07 | 0.13 | 0.21 | 0.11 | 0.14 | -0.35^**^ | -0.25 | 0.64^***^ | -0.49^***^ | 1 |  |  |  |  |
| 11. Sleep Disturbance | -0.20 | 0.03 | -0.15 | -0.17 | -0.01 | 0.36^**^ | 0.18 | -0.72^***^ | 0.63^***^ | -0.73^***^ | 1 |  |  |  |
| 12. General Distress | 0.10 | 0.01 | -0.13 | 0.09 | -0.03 | 0.19 | 0.24 | -0.24 | 0.07 | -0.24 | 0.28^*^ | 1 |  |  |
| 13. Anhedonia | -0.14 | 0.00 | -0.16 | 0.19 | -0.05 | 0.39^**^ | 0.28^*^ | -0.10 | 0.09 | -0.18 | 0.15 | 0.64^***^ | 1 |  |
| 14. Anxious Arousal | -0.06 | 0.03 | 0.23 | -0.08 | 0.01 | -0.02 | -0.13 | -0.18 | -0.04 | -0.13 | 0.25 | 0.08 | -0.06 | 1 |
| *Note.* MEQ, the Morningness-Eveningness Questionnaire, where higher composite scores indicate preferences for morningness. MPS, midpoint of sleep. PVT, the Psychomotor Vigilance Test, where slower reaction times correspond to lower behavioral alertness. SSS, the Stanford Sleepiness Scale, where higher ratings indicate lower subjective alertness. TST, total sleep time. SOL, sleep onset latency. SE, sleep efficiency. Significant effects after the two-stage controlling procedure for the false discovery rate are in bold: ^*^, <.05, ^**^, <.01, ^***^ <.001. | | | | | | | | | | | | | | |

**Table S5**

*Bivariate Correlations for Key Study Variables in Intermediate Chronotypes*

|  | 1 | 2 | 3 | 4 | 5 | 6 | 7 | 8 | 9 | 10 | 11 | 12 | 13 | 14 |
| --- | --- | --- | --- | --- | --- | --- | --- | --- | --- | --- | --- | --- | --- | --- |
| 1. MEQ-Composite | 1 |  |  |  |  |  |  |  |  |  |  |  |  |  |
| 2. MPS | -0.30^***^ | 1 |  |  |  |  |  |  |  |  |  |  |  |  |
| 3. Age | -0.07 | 0.13^*^ | 1 |  |  |  |  |  |  |  |  |  |  |  |
| 4. Morning PVT | -0.07 | 0.03 | 0.00 | 1 |  |  |  |  |  |  |  |  |  |  |
| 5. Evening PVT | 0.05 | -0.01 | -0.04 | 0.48^***^ | 1 |  |  |  |  |  |  |  |  |  |
| 6. Morning SSS | -0.23^***^ | 0.05 | 0.03 | 0.04 | 0.03 | 1 |  |  |  |  |  |  |  |  |
| 7. Evening SSS | 0.12^*^ | -0.15^**^ | -0.14^**^ | 0.04 | 0.21^***^ | 0.30^***^ | 1 |  |  |  |  |  |  |  |
| 8. TST | 0.03 | 0.10 | 0.12^*^ | -0.06 | -0.04 | -0.01 | -0.04 | 1 |  |  |  |  |  |  |
| 9. SOL | -0.03 | -0.14^*^ | -0.04 | 0.13^*^ | 0.08 | 0.20^***^ | 0.11 | -0.20^***^ | 1 |  |  |  |  |  |
| 10. SE | 0.04 | 0.06 | -0.05 | -0.09 | -0.05 | -0.20^***^ | -0.18^**^ | 0.45^***^ | -0.44^***^ | 1 |  |  |  |  |
| 11. Sleep Disturbance | -0.06 | -0.09 | 0.03 | 0.07 | 0.03 | 0.25^***^ | 0.26^***^ | -0.61^***^ | 0.59^***^ | -0.69^***^ | 1 |  |  |  |
| 12. General Distress | -0.11^*^ | 0.06 | -0.13^*^ | 0.02 | -0.01 | 0.29^***^ | 0.35^***^ | -0.15^**^ | 0.13^*^ | -0.22^***^ | 0.35^***^ | 1 |  |  |
| 13. Anhedonia | -0.13^*^ | -0.04 | -0.13^*^ | 0.09 | -0.01 | 0.23^***^ | 0.33^***^ | -0.20^***^ | 0.12^*^ | -0.17^**^ | 0.34^***^ | 0.54^***^ | 1 |  |
| 14. Anxious Arousal | -0.01 | 0.04 | 0.01 | -0.03 | -0.03 | 0.20^***^ | 0.18^**^ | -0.14^**^ | 0.25^***^ | -0.31^***^ | 0.35^***^ | 0.41^***^ | 0.21^***^ | 1 |
| *Note.* MEQ, the Morningness-Eveningness Questionnaire, where higher composite scores indicate preferences for morningness. MPS, midpoint of sleep. PVT, the Psychomotor Vigilance Test, where slower reaction times correspond to lower behavioral alertness. SSS, the Stanford Sleepiness Scale, where higher ratings indicate lower subjective alertness. TST, total sleep time. SOL, sleep onset latency. SE, sleep efficiency. Significant effects after the two-stage controlling procedure for the false discovery rate are in bold: ^*^, <.05, ^**^, <.01, ^***^ <.001. | | | | | | | | | | | | | | |

**Table S6**

*Bivariate Correlations for Key Study Variables in Morning Chronotypes*

|  | 1 | 2 | 3 | 4 | 5 | 6 | 7 | 8 | 9 | 10 | 11 | 12 | 13 | 14 |
| --- | --- | --- | --- | --- | --- | --- | --- | --- | --- | --- | --- | --- | --- | --- |
| 1. MEQ-Composite | 1 |  |  |  |  |  |  |  |  |  |  |  |  |  |
| 2. MPS | -0.47^***^ | 1 |  |  |  |  |  |  |  |  |  |  |  |  |
| 3. Age | 0.15^*^ | -0.02 | 1 |  |  |  |  |  |  |  |  |  |  |  |
| 4. Morning PVT | 0.01 | -0.09 | 0.11 | 1 |  |  |  |  |  |  |  |  |  |  |
| 5. Evening PVT | 0.03 | -0.14^*^ | 0.03 | 0.58^***^ | 1 |  |  |  |  |  |  |  |  |  |
| 6. Morning SSS | -0.22^***^ | 0.04 | -0.07 | 0.03 | 0.02 | 1 |  |  |  |  |  |  |  |  |
| 7. Evening SSS | -0.03 | -0.11 | -0.10 | -0.10 | 0.02 | 0.29^***^ | 1 |  |  |  |  |  |  |  |
| 8. TST | 0.11 | 0.02 | -0.05 | 0.08 | 0.02 | -0.13^*^ | -0.16^**^ | 1 |  |  |  |  |  |  |
| 9. SOL | -0.17^**^ | 0.07 | 0.05 | 0.10 | 0.04 | 0.15^*^ | 0.13^*^ | -0.28^***^ | 1 |  |  |  |  |  |
| 10. SE | 0.15^*^ | -0.04 | -0.12 | 0.03 | 0.01 | -0.29^***^ | -0.20^**^ | 0.53^***^ | -0.43^***^ | 1 |  |  |  |  |
| 11. Sleep Disturbance | -0.15^*^ | 0.01 | 0.09 | 0.02 | -0.03 | 0.28^***^ | 0.26^***^ | -0.66^***^ | 0.55^***^ | -0.68^***^ | 1 |  |  |  |
| 12. General Distress | -0.02 | -0.14^*^ | -0.16^**^ | -0.02 | -0.04 | 0.16^**^ | 0.26^***^ | -0.14^*^ | 0.13^*^ | -0.14^*^ | 0.35^***^ | 1 |  |  |
| 13. Anhedonia | 0.01 | -0.21^**^ | -0.14^*^ | 0.08 | 0.05 | 0.24^***^ | 0.19^**^ | -0.14^*^ | 0.14^*^ | -0.20^**^ | 0.32^***^ | 0.53^***^ | 1 |  |
| 14. Anxious Arousal | -0.09 | 0.00 | -0.08 | 0.00 | 0.00 | 0.29^***^ | 0.25^***^ | -0.24^***^ | 0.09 | -0.24^***^ | 0.37^***^ | 0.43^***^ | 0.26^***^ | 1 |
| *Note.* MEQ, the Morningness-Eveningness Questionnaire, where higher composite scores indicate preferences for morningness. MPS, midpoint of sleep. PVT, the Psychomotor Vigilance Test, where slower reaction times correspond to lower behavioral alertness. SSS, the Stanford Sleepiness Scale, where higher ratings indicate lower subjective alertness. TST, total sleep time. SOL, sleep onset latency. SE, sleep efficiency. Significant effects after the two-stage controlling procedure for the false discovery rate are in bold: ^*^, <.05, ^**^, <.01, ^***^ <.001. | | | | | | | | | | | | | | |

# III. Sleep and Internalizing Symptoms

**Table S7**

*Results for 3 (MEQ: Evening, Intermediate, Morning) x 2 (Age: Middle Age [35-59], Older [60 and Older]) Between-Subjects ANOVAs for Sleep and Internalizing Symptoms*

| **Predictors** | **Sleep** | **Internalizing Symptoms** |
| --- | --- | --- |
|  | ***Total Sleep Time*** | ***General Distress*** |
| MEQ | *F*(2, 646)=1.78, *p*=.169, *ω^2^p*=0.00 | *F*(2, 646)=11.71, *p*<.001, *ω^2^p*=0.03 |
| Age | *F*(1, 646)=3.25, *p*=.072, *ω^2^p*=0.00 | *F*(1, 646)=14.03, *p*<.001, *ω^2^p*=0.02 |
| MEQ x Age | *F*(2, 646)=1.90, *p*=.150, *ω^2^p*=0.00 | *F*(2, 646)=0.34, *p*=.711, *ω^2^p*=0.00 |
|  | ***Sleep Onset Latency*** | ***Anhedonia*** |
| MEQ | *F*(2, 646)=6.82, *p*=.001, *ω^2^p*=0.02 | *F*(2, 646)=20.09, *p*<.001, *ω^2^p*=0.06 |
| Age | *F*(1, 646)=0.08, *p*=.778, *ω^2^p*=0.00 | *F*(1, 646)=7.28, *p*=.007, *ω^2^p*=0.01 |
| MEQ x Age | *F*(2, 646)=1.14, *p*=.320, *ω^2^p*=0.00 | *F*(2, 646)=0.34, *p*=.709, *ω^2^p*=0.00 |
|  | ***Sleep Efficiency*** | ***Anxious Arousal*** |
| MEQ | *F*(2, 646)=1.58, *p*=.206, *ω^2^p*=0.00 | *F*(2, 646)=5.10, *p*=.006, *ω^2^p*=0.01 |
| Age | *F*(1, 646)=1.39, *p*=.239, *ω^2^p*=0.00 | *F*(1, 646)=0.12, *p*=.725, *ω^2^p*=0.00 |
| MEQ x Age | *F*(2, 646)=3.28, *p*=.038, *ω^2^p*=0.01 | *F*(2, 646)=2.11, *p*=.122, *ω^2^p*=0.00 |
|  | ***Sleep Disturbance*** |  |
| MEQ | *F*(2, 646)=7.56, *p*=.001, *ω^2^p*=0.02 |  |
| Age | *F*(1, 646)=0.20, *p*=.657, *ω^2^p*=0.00 |  |
| MEQ x Age | *F*(2, 646)=1.51, *p*=.221, *ω^2^p*=0.00 |  |
| *Note.* MEQ, the Morningness-Eveningness Questionnaire. *ω^2^p*, partial omega-squared, where values were considered small (0.01), medium (0.06), and large (0.14). | | |

**Table S8**

*Follow-Up Pairwise T-Tests Comparing Three MEQ Subtypes (Evening, Intermediate, Morning) for Sleep and Internalizing Symptoms*

| MEQ 1 | MEQ 2 | *T*-*t*es*t* | *p* | *p*.adj | *d* |
| --- | --- | --- | --- | --- | --- |
| **Sleep Onset Latency** | | | | | |
| Evening (M=27.23) | Intermediate (*M=*22.07) | *t*(71)=1.55 | .125 | 1.00 | 0.24 |
| Evening (*M=*27.23) | Morning (*M=*18.52) | *t*(69)=2.64 | .010 | .240 | 0.43 |
| Intermediate (*M=*22.07) | Morning (*M=*18.52) | *t*(589)=2.62 | .009 | .216 | 0.21 |
| **Sleep Efficiency*: Middle Age*** | | | | | |
| Evening (*M=*0.88) | Intermediate (*M=*0.89) | *t*(59)=-0.53 | .597 | 1.00 | -0.09 |
| Evening (*M=*0.88) | Morning (*M=*0.91) | *t*(65)=-1.52 | .134 | 1.00 | -0.27 |
| Intermediate (*M=*0.89) | Morning (*M=*0.91) | *t*(302)=-1.99 | .048 | .576 | -0.21 |
| **Sleep Efficiency*: Older*** | | | | | |
| Evening (*M=*0.95) | Intermediate (*M=*0.87) | *t*(17)=3.59 | .002 | .024 | 0.93 |
| Evening (*M=*0.95) | Morning (*M=*0.89) | *t*(21)=2.77 | .011 | .132 | 0.61 |
| Intermediate (*M=*0.87) | Morning (*M=*0.89) | *t*(210)=-0.89 | .376 | 1.00 | -0.12 |
| **Sleep Disturbance** | | | | | |
| Evening (*M=*6.60) | Intermediate (*M=*5.72) | *t*(81)=1.91 | .060 | 1.00 | 0.27 |
| Evening (*M=*6.60) | Morning (*M=*4.98) | *t*(87)=3.44 | .001 | .024 | 0.50 |
| Intermediate (*M=*5.72) | Morning (*M=*4.98) | *t*(553)=2.77 | .006 | .144 | 0.23 |
| **General Distress** | | | | | |
| Evening (*M=*13.27) | Intermediate (*M=*12.18) | *t*(79)=1.47 | .147 | 1.00 | 0.21 |
| Evening (*M=*13.27) | Morning (*M=*10.68) | *t*(73)=3.55 | .001 | .024 | 0.56 |
| Intermediate (*M=*12.18) | Morning (*M=*10.68) | *t*(589)=4.14 | <.001 | <.001 | 0.34 |
| **Anhedonia** | | | | | |
| Evening (*M=*24.12) | Intermediate (*M=*21.62) | *t*(81)=2.89 | .005 | .120 | 0.41 |
| Evening (*M=*24.12) | Morning (*M=*19.32) | *t*(85)=5.47 | <.001 | <.001 | 0.80 |
| Intermediate (*M=*21.62) | Morning (*M=*19.32) | *t*(560)=4.67 | <.001 | <.001 | 0.39 |
| **Anxious Arousal** | | | | | |
| Evening (*M=*11.50) | Intermediate (*M=*11.60) | *t*(88)=-0.36 | .723 | 1.00 | -0.05 |
| Evening (*M=*11.50) | Morning (*M=*11.06) | *t*(80)=1.55 | .126 | 1.00 | 0.23 |
| Intermediate (*M=*11.60) | Morning (*M=*11.06) | *t*(590)=3.27 | .001 | .024 | 0.27 |
| *Note.* MEQ, the Morningness-Eveningness Questionnaire. *p*.adj, *p*-values were adjusted using the Bonferroni correction. Middle age, 35 to 59. Older, 60 and older. Welch’s approximation for unequal variances was applied. | | | | | |

# IV. Behavioral and Subjective Alertness

**Table S9**

*Results for 3 (Chronotype: Evening, Intermediate, Morning) x 2 (Age: Middle Age [35-59], Older [60 and Older]) x 2 (Time of Day: 7-11 AM, 7-11 PM) Mixed-Effects ANOVAs for Behavioral and Subjective Alertness*

| **Predictors** | **Behavioral Alertness** | **Subjective Alertness** |
| --- | --- | --- |
| MEQ | *F*(2, 646)=0.65, *p*=.524, *ω^2^p*=0.00 | *F*(2, 646)=3.90, *p*=.021, *ω^2^p*=0.01 |
| Age | *F*(1, 646)=2.11, *p*=.147, *ω^2^p*=0.00 | *F*(1, 646)=11.66, *p*=.001, *ω^2^p*=0.02 |
| Time | *F*(1, 646)=0.09, *p*=.769, *ω^2^p*=0.00 | *F*(1, 646)=1.13, *p*=.288, *ω^2^p*=0.00 |
| MEQ x Age | *F*(2, 646)=0.78, *p*=.457, *ω^2^p*=0.00 | *F*(2, 646)=1.91, *p*=.149, *ω^2^p*=0.00 |
| MEQ x Time | *F*(2, 646)=4.20, *p*=.015, *ω^2^p*=0.01 | *F*(2, 646)=17.67, *p<*.001, *ω^2^p*=0.05 |
| Age x Time | *F*(1, 646)=3.32, *p*=.069, *ω^2^p*=0.00 | *F*(1, 646)=4.68, *p*=.031, *ω^2^p*=0.01 |
| MEQ x Age x Time | *F*(2, 646)=1.64, *p*=.195, *ω^2^p*=0.00 | *F*(2, 646)=3.11, *p*=.045, *ω^2^p*=0.01 |
| *Note.* MEQ, the Morningness-Eveningness Questionnaire. *ω^2^p*, partial omega-squared, where values were considered small (0.01), medium (0.06), and large (0.14). | | |

**Table S10**

*Follow-Up Pairwise T-Tests Comparing Three MEQ Subtypes (Evening, Intermediate, Morning) in the Morning (7-11 AM) and Evening (7-11 PM) for Behavioral and Subjective Alertness*

| MEQ 1 | MEQ 2 | *T*-*t*es*t* | *p* | *p*.adj | *d* |
| --- | --- | --- | --- | --- | --- |
| ***Behavioral Alertness*** | | | | | |
| ***7-11 AM*** |  |  |  |  |  |
| Evening (*M=*332.30) | Intermediate (*M=*339.91) | *t*(78)=-0.74 | .464 | 1.00 | -0.11 |
| Evening (*M=*332.30) | Morning (*M=*334.86) | *t*(86)=-0.24 | .810 | 1.00 | -0.04 |
| Intermediate (*M=*339.91) | Morning (*M=*334.86) | *t*(537)=0.87 | .384 | 1.00 | 0.07 |
| ***7-11 PM*** |  |  |  |  |  |
| Evening (*M=*321.39) | Intermediate (*M=*349.32) | *t*(111)=-3.26 | .001 | .012 | -0.39 |
| Evening (*M=*321.39) | Morning (*M=*346.49) | *t*(106)=-2.96 | .004 | .048 | -0.40 |
| Intermediate (*M=*349.32) | Morning (*M=*346.49) | *t*(584)=0.45 | .654 | 1.00 | 0.04 |
| ***AM vs. PM*** |  |  |  |  |  |
| Evening (*M=*332.30 vs. *M=*321.39) | | *t*(59)=1.22 | .229 | 1.00 | 0.16 |
| Intermediate (*M=*339.91 vs. *M=*349.32) | | *t*(335)=-2.20 | .029 | .174 | -0.12 |
| Morning (*M=*334.86 vs. *M=*346.49) | | *t*(255)=-2.89 | .004 | .024 | -0.18 |
| ***Subjective Alertness*** | | | | | |
| ***7-11 AM*** |  |  |  |  |  |
| Evening (*M=*2.78) | Intermediate (*M=*2.02) | *t*(75)=4.78 | <.001 | <.001 | 0.70 |
| Evening (*M=*2.78) | Morning (*M=*1.64) | *t*(76)=7.15 | <.001 | <.001 | 1.11 |
| Intermediate (*M=*2.02) | Morning (*M=*1.64) | *t*(578)=4.90 | <.001 | <.001 | 0.40 |
| ***7-11 PM*** |  |  |  |  |  |
| Evening (*M=*2.40) | Intermediate (*M=*2.32) | *t*(83)=0.46 | .650 | 1.00 | 0.06 |
| Evening (*M=*2.40) | Morning (*M=*2.32) | *t*(91)=0.40 | .691 | 1.00 | 0.06 |
| Intermediate (*M=*2.32) | Morning (*M=*2.32) | *t*(550)=-0.08 | .938 | 1.00 | -0.01 |
| ***AM vs. PM*** |  |  |  |  |  |
| Evening (*M=*2.78 vs. *M=*2.40) | | *t*(59)=1.90 | .062 | .372 | 0.25 |
| Intermediate (*M=*2.02 vs. *M=*2.32) | | *t*(335)=-3.80 | <.001 | <.001 | -0.21 |
| Morning (*M=*1.64 vs. *M=*2.32) | | *t*(255)=-7.90 | <.001 | <.001 | -0.49 |
| *Note.* MEQ, the Morningness-Eveningness Questionnaire. *p*.adj, *p*-values were adjusted using the Bonferroni correction. Welch’s approximation for unequal variances was applied. | | | | | |

**Table S11**

*Follow-Up Pairwise T-Tests Comparing Three MEQ Subtypes (Evening, Intermediate, Morning) and Time of Day (7-11 AM, 7-11 PM) in Middle-Aged and Older Adults for Subjective Alertness*

| MEQ 1 | MEQ 2 | *T*-*t*es*t* | *p* | *p*.adj | *d* |
| --- | --- | --- | --- | --- | --- |
| ***Subjective Alertness: Middle Age*** | | | | | |
| ***7-11 AM*** |  |  |  |  |  |
| Evening (*M=*2.85) | Intermediate (*M=*2.01) | *t*(61)=4.48 | <.001 | <.001 | 0.76 |
| Evening (*M=*2.85) | Morning (*M=*1.76) | *t*(68)=5.67 | <.001 | <.001 | 1.00 |
| Intermediate (*M=*2.01) | Morning (*M=*1.76) | *t*(304)=2.47 | .014 | .168 | 0.26 |
| ***7-11 PM*** |  |  |  |  |  |
| Evening (*M=*2.65) | Intermediate (*M=*2.43) | *t*(70)=1.05 | .299 | 1.00 | 0.16 |
| Evening (*M=*2.65) | Morning (*M=*2.39) | *t*(84)=1.14 | .256 | 1.00 | 0.19 |
| Intermediate (*M=*2.43) | Morning (*M=*2.39) | *t*(298)=0.23 | .820 | 1.00 | 0.02 |
| ***AM vs. PM*** |  |  |  |  |  |
| Evening (*M=*2.85 vs. *M=*2.65) | | *t*(47)=0.88 | .382 | 1.00 | 0.13 |
| Intermediate (*M=*2.01 vs. *M=*2.43) | | *t*(229)=-4.29 | <.001 | <.001 | -0.28 |
| Morning (*M=*1.76 vs. *M=*2.39) | | *t*(139)=-5.19 | <.001 | <.001 | -0.44 |
| ***Subjective Alertness: Older*** | | | | | |
| ***7-11 AM*** |  |  |  |  |  |
| Evening (*M=*2.50) | Intermediate (*M=*2.03) | *t*(14)=1.69 | .113 | 1.00 | 0.49 |
| Evening (*M=*2.50) | Morning (*M=*1.49) | *t*(13)=3.72 | .003 | .036 | 1.20 |
| Intermediate (*M=*2.03) | Morning (*M=*1.49) | *t*(194)=4.36 | <.001 | <.001 | 0.59 |
| ***7-11 PM*** |  |  |  |  |  |
| Evening (*M=*1.42) | Intermediate (*M=*2.08) | *t*(19)=-2.52 | .021 | .252 | -0.61 |
| Evening (*M=*1.42) | Morning (*M=*2.24) | *t*(19)=-3.15 | .005 | .060 | -0.74 |
| Intermediate (*M=*2.08) | Morning (*M=*2.24) | *t*(220)=-0.92 | .357 | 1.00 | -0.12 |
| ***AM vs. PM*** |  |  |  |  |  |
| Evening (*M=*2.50 vs. *M=*1.42) | | *t*(11)=3.77 | .003 | .018 | 1.09 |
| Intermediate (*M=*2.03 vs. *M=*2.08) | | *t*(105)=-0.36 | .721 | 1.00 | -0.03 |
| Morning (*M=*1.49 vs. *M=*2.24) | | *t*(115)=-6.10 | <.001 | <.001 | -0.57 |
| *Note.* MEQ, the Morningness-Eveningness Questionnaire. *p*.adj, *p*-values were adjusted using the Bonferroni correction. Middle age, 35 to 59. Older, 60 and older. Welch’s approximation for unequal variances was applied. | | | | | |

# V. Multivariate Regression Models

**Table S12**

*Standardized Multivariate Regression Results of Chronotype and Age Interacting to Predict Alertness, Sleep, and Internalizing Symptoms*

| Outcome | Predictor | *ß with 95% CI* | *S.E.* | *p* | *p.adj* |
| --- | --- | --- | --- | --- | --- |
| **Morning PVT** | MEQ-Composite | -0.07 (-0.42, 0.27) | 0.17 | .676 | .654 |
|  | Age | 0.04 (-0.17, 0.25) | 0.11 | .714 | .668 |
|  | Interaction | 0.05 (-0.38, 0.48) | 0.22 | .826 | .749 |
| **Evening PVT** | MEQ-Composite | -0.02 (-0.36, 0.33) | 0.17 | .927 | .814 |
|  | Age | -0.06 (-0.28, 0.15) | 0.11 | .562 | .582 |
|  | Interaction | 0.10 (-0.33, 0.53) | 0.22 | .641 | .641 |
| **Morning SSS** | MEQ-Composite | -0.59 (-0.90, -0.28) | 0.16 | <.001 | .007 |
|  | Age | -0.15 (-0.35, 0.05) | 0.10 | .141 | .186 |
|  | Interaction | 0.28 (-0.11, 0.68) | 0.20 | .162 | .204 |
| **Evening SSS** | MEQ-Composite | -0.18 (-0.52, 0.16) | 0.17 | .295 | .343 |
|  | Age | -0.28 (-0.49, -0.07) | 0.11 | .009 | .045 |
|  | Interaction | 0.28 (-0.14, 0.71) | 0.22 | .190 | .229 |
| **PSQI-TST** | MEQ-Composite | 0.42 (0.08, 0.76) | 0.17 | .015 | .061 |
|  | Age | 0.25 (0.04, 0.46) | 0.11 | .021 | .074 |
|  | Interaction | -0.42 (-0.85, 0.00) | 0.22 | .050 | .113 |
| **PSQI-SOL** | MEQ-Composite | -0.46 (-0.79, -0.12) | 0.17 | .007 | .043 |
|  | Age | -0.19 (-0.40, 0.02) | 0.11 | .071 | .130 |
|  | Interaction | 0.38 (-0.04, 0.80) | 0.21 | .076 | .130 |
| **PSQI-SE** | MEQ-Composite | 0.37 (0.03, 0.70) | 0.17 | .034 | .090 |
|  | Age | 0.10 (-0.11, 0.32) | 0.11 | .339 | .378 |
|  | Interaction | -0.34 (-0.77, 0.08) | 0.22 | .112 | .171 |
| **PSQI-Composite** | MEQ-Composite | -0.56 (-0.89, -0.22) | 0.17 | .001 | .015 |
|  | Age | -0.17 (-0.38, 0.03) | 0.11 | .102 | .165 |
|  | Interaction | 0.47 (0.05, 0.88) | 0.21 | .029 | .084 |
| **General Distress** | MEQ-Composite | -0.26 (-0.59, 0.07) | 0.17 | .125 | .181 |
|  | Age | -0.20 (-0.41, 0.01) | 0.11 | .059 | .114 |
|  | Interaction | 0.13 (-0.29, 0.55) | 0.21 | .547 | .582 |
| **Anhedonia** | MEQ-Composite | -0.53 (-0.85, -0.20) | 0.17 | .002 | .015 |
|  | Age | -0.32 (-0.52, -0.12) | 0.10 | .002 | .015 |
|  | Interaction | 0.40 (-0.01, 0.80) | 0.21 | .058 | .114 |
| **Anxious Arousal** | MEQ-Composite | 0.26 (-0.08, 0.60) | 0.17 | .137 | .186 |
|  | Age | 0.22 (0.01, 0.44) | 0.11 | .039 | .093 |
|  | Interaction | -0.48 (-0.90, -0.05) | 0.22 | .027 | .084 |
| *Note.* MEQ, the Morningness-Eveningness Questionnaire, where higher composite scores indicate preferences for morningness. PSQI, the Pittsburgh Sleep Quality Index, where higher composite scores represent greater sleep disturbance. PVT, the Psychomotor Vigilance Test, where slower reaction times correspond to lower behavioral alertness. SSS, the Stanford Sleepiness Scale, where higher ratings indicate lower subjective alertness. TST, total sleep time. SOL, sleep onset latency. SE, sleep efficiency. *p.adj*, *p*-values after controlling for false discovery rates. | | | | | |

**Table S13**

*Standardized Multivariate Regression Results of Chronotype and Age Predicting Alertness, Sleep, and Internalizing Symptoms in Evening, Intermediate, and Morning Chronotypes*

| Outcome | Predictor | *ß (SE)* | *p.adj* | *ß (SE)* | *p.adj* | *ß (SE)* | *p.adj* |
| --- | --- | --- | --- | --- | --- | --- | --- |
|  |  | **Evening (*n*=60)** | | **Intermediate (*n*=336)** | | **Morning (*n*=256)** | |
| **Morning PVT** | MEQ-Composite | -0.01 (0.06) | .902 | -0.07 (0.05) | .433 | 0.01 (0.13) | .902 |
|  | Age | 0.11 (0.06) | .202 | -0.00 (0.05) | .928 | 0.14 (0.13) | .530 |
| **Evening PVT** | MEQ-Composite | 0.02 (0.06) | .813 | 0.05 (0.05) | .610 | -0.13 (0.13) | .534 |
|  | Age | 0.03 (0.06) | .743 | -0.04 (0.05) | .665 | -0.02 (0.13) | .900 |
| **Morning SSS** | MEQ-Composite | -0.21 (0.06) | .012 | -0.23 (0.05) | <.001 | -0.30 (0.11) | .052 |
|  | Age | -0.03 (0.06) | .707 | 0.01 (0.05) | .900 | -0.15 (0.12) | .484 |
| **Evening SSS** | MEQ-Composite | -0.02 (0.06) | .886 | 0.11 (0.05) | .146 | -0.07 (0.12) | .707 |
|  | Age | -0.10 (0.06) | .329 | -0.13 (0.05) | .061 | -0.31 (0.11) | .052 |
| **PSQI-TST** | MEQ-Composite | 0.12 (0.06) | .175 | 0.04 (0.05) | .665 | 0.22 (0.12) | .202 |
|  | Age | -0.07 (0.06) | .530 | 0.12 (0.05) | .109 | 0.10 (0.12) | .610 |
| **PSQI-SOL** | MEQ-Composite | -0.18 (0.06) | .052 | -0.04 (0.05) | .665 | -0.07 (0.13) | .707 |
|  | Age | 0.08 (0.06) | .492 | -0.05 (0.05) | .610 | -0.08 (0.13) | .687 |
| **PSQI-SE** | MEQ-Composite | 0.17 (0.06) | .052 | 0.04 (0.05) | .665 | 0.05 (0.13) | .813 |
|  | Age | -0.15 (0.06) | .083 | -0.05 (0.05) | .610 | 0.20 (0.12) | .265 |
| **PSQI-Composite** | MEQ-Composite | -0.17 (0.06) | .052 | -0.06 (0.05) | .530 | -0.19 (0.12) | .329 |
|  | Age | 0.12 (0.06) | .171 | 0.02 (0.05) | .756 | -0.13 (0.12) | .534 |
| **General Distress** | MEQ-Composite | 0.01 (0.06) | .902 | -0.12 (0.05) | .109 | 0.12 (0.13) | .572 |
|  | Age | -0.16 (0.06) | .052 | -0.14 (0.05) | .052 | -0.14 (0.13) | .530 |
| **Anhedonia** | MEQ-Composite | 0.03 (0.06) | .743 | -0.14 (0.05) | .059 | -0.13 (0.13) | .537 |
|  | Age | -0.14 (0.06) | .107 | -0.14 (0.05) | .052 | -0.14 (0.13) | .529 |
| **Anxious Arousal** | MEQ-Composite | -0.08 (0.06) | .450 | -0.01 (0.05) | .900 | -0.09 (0.13) | .665 |
|  | Age | -0.06 (0.06) | .534 | 0.01 (0.05) | .902 | 0.24 (0.12) | .169 |
| *Note.* MEQ, the Morningness-Eveningness Questionnaire, where higher composite scores indicate preferences for morningness. PSQI, the Pittsburgh Sleep Quality Index, where higher composite scores represent greater sleep disturbance. PVT, the Psychomotor Vigilance Test, where slower reaction times correspond to lower behavioral alertness. SSS, the Stanford Sleepiness Scale, where higher ratings indicate lower subjective alertness. TST, total sleep time. SOL, sleep onset latency. SE, sleep efficiency. *p.adj*, *p*-values after controlling for false discovery rates. | | | | | | | |

**Table S14**

*Standardized Multivariate Regression Results of Chronotype and Age Predicting Alertness, Sleep, and Internalizing Symptoms in Middle-Aged (35-59) and Older Adults (60 and Above)*

| Outcome | Predictor | *ß (SE)* | *p.adj* | *ß (SE)* | *p.adj* |  |
| --- | --- | --- | --- | --- | --- | --- |
|  |  | **Middle-Aged (*n*=418)** | | **Older (*n*=234)** | |  |
| **Morning PVT** | MEQ-Composite | -0.11 (0.06) | .157 | 0.02 (0.05) | .631 |  |
|  | Age | 0.11 (0.06) | .173 | 0.00 (0.05) | .771 |  |
| **Evening PVT** | MEQ-Composite | 0.04 (0.07) | .549 | 0.08 (0.05) | .173 |  |
|  | Age | 0.08 (0.06) | .336 | -0.09 (0.05) | .151 |  |
| **Morning SSS** | MEQ-Composite | -0.38 (0.05) | <.001 | -0.36 (0.04) | <.001 |  |
|  | Age | 0.03 (0.06) | .631 | 0.04 (0.05) | .455 |  |
| **Evening SSS** | MEQ-Composite | 0.12 (0.06) | .128 | -0.01 (0.05) | .686 |  |
|  | Age | -0.14 (0.06) | .064 | -0.06 (0.05) | .334 |  |
| **PSQI-TST** | MEQ-Composite | 0.00 (0.07) | .771 | 0.15 (0.05) | .011 |  |
|  | Age | -0.05 (0.07) | .500 | 0.01 (0.05) | .691 |  |
| **PSQI-SOL** | MEQ-Composite | -0.08 (0.06) | .334 | -0.20 (0.05) | <.001 |  |
|  | Age | 0.01 (0.07) | .697 | -0.02 (0.05) | .631 |  |
| **PSQI-SE** | MEQ-Composite | 0.04 (0.06) | .553 | 0.13 (0.05) | .036 |  |
|  | Age | -0.11 (0.06) | .165 | 0.01 (0.05) | .691 |  |
| **PSQI-Composite** | MEQ-Composite | -0.10 (0.06) | .173 | -0.24 (0.05) | <.001 |  |
|  | Age | 0.03 (0.06) | .631 | 0.05 (0.05) | .340 |  |
| **General Distress** | MEQ-Composite | -0.14 (0.06) | .064 | -0.18 (0.05) | .001 |  |
|  | Age | -0.16 (0.06) | .036 | 0.00 (0.05) | .771 |  |
| **Anhedonia** | MEQ-Composite | -0.15 (0.06) | .053 | -0.26 (0.05) | <.001 |  |
|  | Age | -0.14 (0.06) | .064 | -0.06 (0.05) | .327 |  |
| **Anxious Arousal** | MEQ-Composite | -0.24 (0.06) | <.001 | -0.04 (0.05) | .461 |  |
|  | Age | -0.05 (0.06) | .489 | 0.04 (0.05) | .500 |  |
| *Note.* MEQ, the Morningness-Eveningness Questionnaire, where higher composite scores indicate preferences for morningness. PSQI, the Pittsburgh Sleep Quality Index, where higher composite scores represent greater sleep disturbance. PVT, the Psychomotor Vigilance Test, where slower reaction times correspond to lower behavioral alertness. SSS, the Stanford Sleepiness Scale, where higher ratings indicate lower subjective alertness. TST, total sleep time. SOL, sleep onset latency. SE, sleep efficiency. *p.adj*, *p*-values after controlling for false discovery rates. | | | | | | |

# VI. Description of the Larger Projects

We combined data from three larger studies investigating the effects of sleep and aging on emotional memory consolidation (Denis et al., 2022; Niu et al., 2024). All three studies employed a similar design, where participants completed the emotional memory trade-off task (Denis et al., 2022; Niu et al., 2024). To ensure data quality, participants were instructed to complete both experiment sessions using a laptop or desktop computer with a compatible web browser such as Google Chrome or Edge. Attempts to access the experiment using incompatible devices (e.g., mobile phones, tablets, or Internet Explorer) resulted in an error message that prompted participants to use an appropriate device. During incidental encoding, they were presented with a series of emotional and neutral scenes, each featuring an emotional or neutral object placed on an always neutral background. After a 12-hour delay, participants had a surprise memory test where they indicated whether each object or background was old or new compared to what they viewed during encoding. Before both encoding and retrieval, all participants performed a brief 3-min version of the Psychomotor Vigilance Test (Basner et al., 2011) and responded to the Standford Sleepiness Scale (Shahid et al., 2012). The procedures were identical across all three studies, except for the valence of the emotional scenes: negative for study 1, positive for study 2, and both positive and negative for study 3.

In all three studies, participants were randomly assigned to either the nighttime sleep or the daytime wake condition. The sleep condition completed the encoding task in the evening (7-11 PM) and memory task the following morning (7-11 AM), while the wake condition encoded in the morning (7-11 AM) and had their memories tested the same evening (7-11 PM). Although the current study was not interested in testing sleep-related influences on memory processing, this design allowed us to assess alertness at different times of day. Additionally, by comparing groups that completed alertness measures in different orders (sleep group: evening-morning vs. wake group: morning-evening), we were able to control for potential order effects.

# VII. Measurement Invariance Testing

We tested for scalar invariance by constraining the intercepts to be equal across session order (morning first, evening first) and experiment (study 1, study 2, study 3). We compared if model fit indices were worse for the intercept-constrained models compared to the intercept-free models. As our analysis did not involve latent variables or factor loadings, metric invariance testing was not applicable. See **Table S15** for a summary of measurement invariance indices. Results indicated that scalar invariance was not established for either session order or experiment, based on both significant differences in model fit indices, and violations of recommended guidelines for measurement invariance in larger samples (*n*>300): ΔCFI<-.010, ΔRMSEA<.015, and ΔSRMR<.010 (Chen, 2007; Cheung & Rensvold, 2002). This indicated that intercepts were different between morning-first and evening-first participants, and among the three experiments. Therefore, we freed the intercepts across session orders as well as the three experiments while conducting multigroup multivariate regression analyses. The overall patterns of the effects of chronotype and age on alertness, sleep, and internalizing symptoms were similar across session orders (**Table S16**) and experiments (**Table S17**).

Since internalizing symptoms were measured between 7–11 AM for the morning-first group and 7–11 PM for the evening-first group, we conducted 3 (Chronotype Group: Evening, Intermediate, Morning) × 2 (Age Group: Middle-Aged [35–59], Older [60+]) × 2 (Time of Day: 7–11 AM, 7–11 PM) mixed-effects ANOVAs on general distress, anhedonia, and anxious arousal. No significant effects were found for time of day (**Table S18**, *ps*>.9).

**Table S15**

*Measurement Invariance Across Session Orders and Experiments*

| Model | CFI | RMSEA | SRMR | AIC | BIC |
| --- | --- | --- | --- | --- | --- |
| **Session order (*n*=322 for evening first, *n*=330 for morning first)** | | | | | |
| Configural model | 1.00 | 0.00 | 0.00 | -7466.77 | -6579.72 |
| Scalar model | 1.00 | 0.05 | 0.02 | -7467.68 | -6629.91 |
| Configural-scalar Δ | -0.00 | 0.05 | 0.02 | -0.91 | -50.19 |
| Chi-square difference | Δ*χ²*(11 )=21.09, *p*=.032 | | | | |
| **Experiment (*n*=184 for study 1, *n*=235 for study 2, *n*=233 for study 3)** | | | | | |
| Configural model | 1.00 | 0.00 | 0.00 | -7540.60 | -6210.02 |
| Scalar model | 0.99 | 0.06 | 0.03 | -7546.78 | -6314.77 |
| Configural-scalar Δ | -0.01 | 0.06 | 0.03 | -6.19 | -104.75 |
| Chi-square difference | Δ*χ²*(22)=37.81, *p* =.019 | | | | |

*Note.* CFI, comparative fit index. RMSEA, root-mean-square error of approximation. SRMR, standardized root mean-squared residual. AIC, Akaike information criterion. BIC, Bayes information criterion.

**Table S16**

*Standardized Multivariate Regression Results of Chronotype and Age Predicting Alertness, Sleep, and Internalizing Symptoms in Evening First and Morning First Participants*

| Outcome | Predictor | *ß (SE)* | *p.adj* | *ß (SE)* | *p.adj* |  |
| --- | --- | --- | --- | --- | --- | --- |
|  |  | **Evening Frist (*n*=322)** | | **Morning First (*n*=330)** | |  |
| **Morning PVT** | MEQ-Composite | -0.05 (0.06) | .320 | -0.03 (0.06) | .433 |  |
|  | Age | 0.17 (0.06) | .007 | -0.00 (0.06) | .563 |  |
| **Evening PVT** | MEQ-Composite | 0.03 (0.06) | .414 | 0.10 (0.05) | .089 |  |
|  | Age | 0.02 (0.06) | .488 | -0.04 (0.06) | .335 |  |
| **Morning SSS** | MEQ-Composite | -0.40 (0.05) | <.001 | -0.35 (0.05) | <.001 |  |
|  | Age | 0.04 (0.05) | .335 | -0.07 (0.05) | .185 |  |
| **Evening SSS** | MEQ-Composite | -0.07 (0.06) | .188 | 0.13 (0.05) | .023 |  |
|  | Age | -0.09 (0.06) | .125 | -0.19 (0.05) | .001 |  |
| **PSQI-TST** | MEQ-Composite | 0.06 (0.06) | .241 | 0.13 (0.05) | .025 |  |
|  | Age | -0.01 (0.06) | .542 | 0.12 (0.05) | .040 |  |
| **PSQI-SOL** | MEQ-Composite | -0.14 (0.06) | .023 | -0.19 (0.05) | .001 |  |
|  | Age | 0.08 (0.06) | .164 | -0.10 (0.05) | .076 |  |
| **PSQI-SE** | MEQ-Composite | 0.07 (0.06) | .211 | 0.15 (0.05) | .013 |  |
|  | Age | -0.12 (0.06) | .043 | 0.02 (0.05) | .484 |  |
| **PSQI-Composite** | MEQ-Composite | -0.14 (0.06) | .023 | -0.25 (0.05) | <.001 |  |
|  | Age | 0.06 (0.06) | .238 | 0.02 (0.05) | .486 |  |
| **General Distress** | MEQ-Composite | -0.16 (0.06) | .010 | -0.17 (0.05) | .004 |  |
|  | Age | -0.12 (0.06) | .048 | -0.17 (0.05) | .004 |  |
| **Anhedonia** | MEQ-Composite | -0.23 (0.05) | <.001 | -0.21 (0.05) | <.001 |  |
|  | Age | -0.05 (0.06) | .324 | -0.22 (0.05) | <.001 |  |
| **Anxious Arousal** | MEQ-Composite | -0.07 (0.06) | .230 | -0.15 (0.05) | .012 |  |
|  | Age | -0.03 (0.06) | .414 | 0.03 (0.05) | .425 |  |
| *Note.* MEQ, the Morningness-Eveningness Questionnaire, where higher composite scores indicate preferences for morningness. PSQI, the Pittsburgh Sleep Quality Index, where higher composite scores represent greater sleep disturbance. PVT, the Psychomotor Vigilance Test, where slower reaction times correspond to lower behavioral alertness. SSS, the Stanford Sleepiness Scale, where higher ratings indicate lower subjective alertness. TST, total sleep time. SOL, sleep onset latency. SE, sleep efficiency. *p.adj*, *p*-values after controlling for false discovery rates. | | | | | | |

**Table S17**

*Standardized Multivariate Regression Results of Chronotype and Age Predicting Alertness, Sleep, and Internalizing Symptoms in the Three Experiments*

| Outcome | Predictor | *ß (SE)* | *p.adj* | *ß (SE)* | *p.adj* | *ß (SE)* | *p.adj* |
| --- | --- | --- | --- | --- | --- | --- | --- |
|  |  | **Experiment 1 (*n*=184)** | | **Experiment 2 (*n*=235)** | | **Experiment 3 (*n*=233)** | |
| **Morning PVT** | MEQ-Composite | -0.05 (0.07) | .621 | -0.05 (0.07) | .561 | -0.02 (0.07) | .770 |
|  | Age | 0.14 (0.07) | .158 | 0.02 (0.07) | .770 | -0.02 (0.07) | .749 |
| **Evening PVT** | MEQ-Composite | 0.10 (0.07) | .273 | 0.03 (0.07) | .742 | 0.09 (0.07) | .273 |
|  | Age | 0.04 (0.07) | .708 | -0.03 (0.07) | .742 | -0.01 (0.07) | .770 |
| **Morning SSS** | MEQ-Composite | -0.33 (0.06) | <.001 | -0.45 (0.05) | <.001 | -0.30 (0.06) | <.001 |
|  | Age | 0.03 (0.07) | .742 | -0.01 (0.06) | .770 | 0.02 (0.06) | .770 |
| **Evening SSS** | MEQ-Composite | 0.09 (0.07) | .336 | 0.06 (0.07) | .530 | 0.00 (0.07) | .833 |
|  | Age | -0.25 (0.07) | .002 | -0.04 (0.07) | .621 | -0.15 (0.06) | .088 |
| **PSQI-TST** | MEQ-Composite | 0.11 (0.07) | .242 | 0.14 (0.07) | .104 | 0.03 (0.07) | .742 |
|  | Age | 0.10 (0.07) | .273 | -0.01 (0.07) | .812 | 0.06 (0.07) | .521 |
| **PSQI-SOL** | MEQ-Composite | -0.12 (0.07) | .205 | -0.17 (0.07) | .053 | -0.17 (0.06) | .044 |
|  | Age | 0.15 (0.07) | .120 | -0.01 (0.07) | .812 | -0.10 (0.06) | .210 |
| **PSQI-SE** | MEQ-Composite | 0.13 (0.07) | .182 | 0.15 (0.07) | .100 | -0.00 (0.07) | .855 |
|  | Age | -0.13 (0.07) | .182 | -0.13 (0.07) | .158 | 0.02 (0.07) | .770 |
| **PSQI-Composite** | MEQ-Composite | -0.18 (0.07) | .058 | -0.27 (0.06) | <.001 | -0.11 (0.07) | .205 |
|  | Age | 0.08 (0.07) | .414 | 0.11 (0.07) | .182 | -0.01 (0.07) | .812 |
| **General Distress** | MEQ-Composite | -0.09 (0.07) | .336 | -0.24 (0.06) | .001 | -0.11 (0.06) | .182 |
|  | Age | -0.16 (0.07) | .088 | -0.08 (0.07) | .336 | -0.15 (0.06) | .088 |
| **Anhedonia** | MEQ-Composite | -0.07 (0.07) | .454 | -0.32 (0.06) | <.001 | -0.21 (0.06) | .003 |
|  | Age | -0.13 (0.07) | .182 | -0.06 (0.06) | .454 | -0.18 (0.06) | .028 |
| **Anxious Arousal** | MEQ-Composite | -0.15 (0.07) | .122 | -0.08 (0.07) | .336 | -0.11 (0.07) | .205 |
|  | Age | -0.06 (0.07) | .536 | 0.02 (0.07) | .749 | 0.03 (0.07) | .742 |
| *Note.* MEQ, the Morningness-Eveningness Questionnaire, where higher composite scores indicate preferences for morningness. PSQI, the Pittsburgh Sleep Quality Index, where higher composite scores represent greater sleep disturbance. PVT, the Psychomotor Vigilance Test, where slower reaction times correspond to lower behavioral alertness. SSS, the Stanford Sleepiness Scale, where higher ratings indicate lower subjective alertness. TST, total sleep time. SOL, sleep onset latency. SE, sleep efficiency. *p.adj*, *p*-values after controlling for false discovery rates. | | | | | | | |

**Table S18**

*Results for 3 (MEQ: Evening, Intermediate, Morning) x 2 (Age: Middle Age [35-59], Older [60 and Older]) x 2 (Time of Day: 7-11 AM, 7-11 PM) Mixed-Effects ANOVAs for Internalizing Symptoms*

| **Predictors** | **General Distress** | **Anhedonia** | **Anxious Arousal** |
| --- | --- | --- | --- |
| MEQ | *F*(2, 640)=11.65, *p<*.001, *ω^2^p*=0.03 | *F*(2, 640)=20.16, *p<*.001, *ω^2^p*=0.06 | *F*(2, 640)=5.08, *p*=.006, *ω^2^p*=0.01 |
| Age | *F*(1, 640)=13.95, *p<*.001, *ω^2^p*=0.02 | *F*(1, 640)=7.31, *p*=.007, *ω^2^p*=0.01 | *F*(1, 640)=0.12, *p*=.726, *ω^2^p*=0.00 |
| Time | *F*(1, 640)=0.05, *p*=.822, *ω^2^p*=0.00 | *F*(1, 640)=0.33, *p*=.567, *ω^2^p*=0.00 | *F*(1, 640)=0.34, *p*=.560, *ω^2^p*=0.00 |
| MEQ x Age | *F*(2, 640)=0.35, *p*=.704, *ω^2^p*=0.00 | *F*(2, 640)=0.36, *p*=.697, *ω^2^p*=0.00 | *F*(2, 640)=2.04, *p*=.131, *ω^2^p*=0.00 |
| MEQ x Time | *F*(2, 640)=1.05, *p*=.349, *ω^2^p*=0.00 | *F*(2, 640)=1.11, *p*=.331, *ω^2^p*=0.00 | *F*(2, 640)=0.60, *p*=.548, *ω^2^p*=0.00 |
| Age x Time | *F*(1, 640)=0.31, *p*=.578, *ω^2^p*=0.00 | *F*(1, 640)=1.81, *p*=.179, *ω^2^p*=0.00 | *F*(1, 640)=0.06, *p*=.808, *ω^2^p*=0.00 |
| MEQ x Age X Time | *F*(2, 640)=0.00, *p*=.998, *ω^2^p*=0.00 | *F*(2, 640)=1.91, *p*=.148, *ω^2^p*=0.00 | *F*(2, 640)=1.01, *p*=.366, *ω^2^p*=0.00 |
| *Note.* MEQ, the Morningness-Eveningness Questionnaire. *ω^2^p*, partial omega-squared, where values were considered small (0.01), medium (0.06), and large (0.14). | | | |

# VIII. Comparing Extreme Values

**Table S19**

*Pairwise T-Tests Comparing Extreme Evening and Morning MEQ Chronotypes for Alertness, Sleep, Internalizing Symptoms*

| Outcome | MEQ 1 | MEQ 2 |  | *T*-*t*es*t* | *p* | *p*.adj | *d* |
| --- | --- | --- | --- | --- | --- | --- | --- |
| Morning PVT | Evening (*M=*341.74) | Morning (*M=*341.96) |  | *t*(16)=-0.01 | .991 | 1.00 | -0.00 |
| Evening PVT | Evening (*M=*347.16) | Morning (*M=*351.05) |  | *t*(7)=-0.11 | .913 | 1.00 | -0.05 |
| Morning SSS | Evening (*M=*3.86) | Morning (*M=*1.34) |  | *t*(7)=7.16 | <.001 | <.001 | 3.13 |
| Evening SSS | Evening (*M=*2.43) | Morning (*M=*2.33) |  | *t*(7)=0.20 | .848 | 1.00 | 0.08 |
| PSQI-TST | Evening (*M=*377.14) | Morning (*M=*433.59) |  | *t*(8)=-3.03 | .016 | .176 | -1.08 |
| PSQI-SOL | Evening (*M=*20.00) | Morning (*M=*14.56) |  | *t*(7)=0.99 | .355 | 1.00 | 0.39 |
| PSQI-SE | Evening (*M=*0.90) | Morning (*M=*0.92) |  | *t*(8)=-0.54 | .603 | 1.00 | -0.19 |
| PSQI-Composite | Evening (*M=*6.71) | Morning (*M=*4.06) |  | *t*(10)=3.60 | .005 | .055 | 1.13 |
| General Distress | Evening (*M=*10.43) | Morning (*M=*10.64) |  | *t*(9)=-0.18 | .863 | 1.00 | -0.06 |
| Anhedonia | Evening (*M=*25.86) | Morning (*M=*19.61) |  | *t*(8)=2.64 | .029 | .319 | 0.97 |
| Anxious Arousal | Evening (*M=*12.86) | Morning (*M=*10.70) |  | *t*(6)=1.67 | .145 | 1.00 | 0.83 |
| *Note.* MEQ, the Morningness-Eveningness Questionnaire. PSQI, the Pittsburgh Sleep Quality Index, where higher composite scores represent greater sleep disturbance. PVT, the Psychomotor Vigilance Test, where slower reaction times correspond to lower behavioral alertness. SSS, the Stanford Sleepiness Scale, where higher ratings indicate lower subjective alertness. TST, total sleep time. SOL, sleep onset latency. SE, sleep efficiency. *p*.adj, *p*-values were adjusted using the Bonferroni correction. Welch’s approximation for unequal variances was applied. | | | | | | | |

**Table S20**

*Pairwise T-Tests Comparing Youngest Middle-Aged Adults (age 35-39) and Oldest Older Adults (age 71-92) for Behavioral and Subjective Alertness*

| Time | Chronotype | Middle | Older | *T*-*t*es*t* | *p* | *p*.adj | *d* |
| --- | --- | --- | --- | --- | --- | --- | --- |
| **Behavioral Alertness** | | | | | | | |
| 7-11 AM | Evening | *M*=336.99 | *M*=336.23 | *t*(21)=0.06 | .950 | 1.00 | 0.02 |
|  | Intermediate | *M*=344.51 | *M*=344.54 | *t*(25)=-0.00 | .999 | 1.00 | -0.00 |
|  | Morning | *M*=311.17 | *M*=339.23 | *t*(21)=-1.34 | .195 | 1.00 | -0.45 |
| 7-11 PM | Evening | *M*=327.19 | *M*=299.22 | *t*(7)=1.83 | .107 | 1.00 | 0.67 |
|  | Intermediate | *M*=360.70 | *M*=352.83 | *t*(29)=0.41 | .688 | 1.00 | 0.11 |
|  | Morning | *M*=338.61 | *M*=346.87 | *t*(37)=-0.38 | .707 | 1.00 | -0.12 |
| **Subjective Alertness** | | | | | | | |
| 7-11 AM | Evening | *M*=3.14 | *M*=3.50 | *t*(2)=-0.63 | .600 | 1.00 | -0.34 |
|  | Intermediate | *M*=2.02 | *M*=2.25 | *t*(15)=-0.58 | .573 | 1.00 | -0.19 |
|  | Morning | *M*=1.46 | *M*=1.69 | *t*(33)=-0.81 | .424 | 1.00 | -0.26 |
| 7-11 PM | Evening | *M*=2.64 | *M*=1.00 | *t*(21)=5.13 | <.001 | <.001 | 1.55 |
|  | Intermediate | *M*=2.49 | *M*=2.08 | *t*(17)=0.96 | .351 | 1.00 | 0.30 |
|  | Morning | *M*=2.38 | *M*=1.75 | *t*(38)=1.59 | .121 | 1.00 | 0.49 |
| *Note.* PVT, the Psychomotor Vigilance Test, where slower reaction times correspond to lower behavioral alertness. SSS, the Stanford Sleepiness Scale, where higher ratings indicate lower subjective alertness. *p*.adj, *p*-values were adjusted using the Bonferroni correction. Welch’s approximation for unequal variances was applied. | | | | | | | |

**Table S21**

*Pairwise T-Tests Comparing Youngest Middle-Aged Adults (age 35-39) and Oldest Older Adults (age 71-92) for Sleep and Internalizing Symptoms*

| Outcome | Chronotype | Middle | Older | *T*-*t*es*t* | *p* | *p*.adj | *d* |
| --- | --- | --- | --- | --- | --- | --- | --- |
| PSQI-TST | Evening | *M*=392.73 | *M*=390.00 | *t*(2)=0.08 | .945 | 1.00 | 0.04 |
|  | Intermediate | *M*=409.53 | *M*=465.00 | *t*(14)=-2.31 | .037 | 1.00 | -0.80 |
|  | Morning | *M*=430.62 | *M*=397.50 | *t*(27)=1.84 | .077 | 1.00 | 0.61 |
| PSQI-SOL | Evening | *M*=29.09 | *M*=5.00 | *t*(21)=4.28 | <.001 | <.001 | 1.29 |
|  | Intermediate | *M*=24.47 | *M*=15.83 | *t*(36)=1.89 | .066 | 1.00 | 0.47 |
|  | Morning | *M*=22.08 | *M*=23.81 | *t*(36)=-0.28 | .780 | 1.00 | -0.09 |
| PSQI-SE | Evening | *M*=0.87 | *M*=0.96 | *t*(3)=-1.92 | .156 | 1.00 | -0.85 |
|  | Intermediate | *M*=0.88 | *M*=0.90 | *t*(20)=-0.61 | .551 | 1.00 | -0.18 |
|  | Morning | *M*=0.91 | *M*=0.85 | *t*(29)=1.84 | .076 | 1.00 | 0.61 |
| PSQI-Composite | Evening | *M*=6.86 | *M*=6.50 | *t*(7)=0.43 | .679 | 1.00 | 0.16 |
|  | Intermediate | *M*=5.51 | *M*=3.92 | *t*(17)=1.63 | .121 | 1.00 | 0.51 |
|  | Morning | *M*=4.54 | *M*=6.38 | *t*(31)=-1.55 | .131 | 1.00 | -0.50 |
| General distress | Evening | *M*=14.27 | *M*=8.50 | *t*(17)=4.55 | <.001 | <.001 | 1.48 |
|  | Intermediate | *M*=13.42 | *M*=9.17 | *t*(59)=4.17 | <.001 | <.001 | 0.91 |
|  | Morning | *M*=11.08 | *M*=9.50 | *t*(28)=1.44 | .161 | 1.00 | 0.42 |
| Anhedonia | Evening | *M*=25.05 | *M*=19.50 | *t*(3)=2.88 | .073 | 1.00 | 1.29 |
|  | Intermediate | *M*=23.04 | *M*=18.83 | *t*(24)=2.54 | .018 | .594 | 0.70 |
|  | Morning | *M*=19.92 | *M*=17.88 | *t*(38)=1.17 | .250 | 1.00 | 0.36 |
| Anxious arousal | Evening | *M*=11.32 | *M*=13.50 | *t*(1)=-0.86 | .542 | 1.00 | -0.79 |
|  | Intermediate | *M*=11.89 | *M*=10.83 | *t*(28)=1.85 | .074 | 1.00 | 0.49 |
|  | Morning | *M*=10.75 | *M*=10.44 | *t*(38)=0.89 | .377 | 1.00 | 0.27 |
| *Note.* PSQI, the Pittsburgh Sleep Quality Index, where higher composite scores represent greater sleep disturbance. TST, total sleep time. SOL, sleep onset latency. SE, sleep efficiency. *p*.adj, *p*-values were adjusted using the Bonferroni correction. Welch’s approximation for unequal variances was applied. | | | | | | | |

# IX. Analyses Including Outliers

**Table S22**

*Results for 3 (MEQ: Evening, Intermediate, Morning) x 2 (Age: Middle Age [35-59], Older [60 and Older]) Between-Subjects ANOVAs for Sleep and Internalizing Symptoms While Including 59 Outliers*

| **Predictors** | **Sleep** | **Internalizing Symptoms** |
| --- | --- | --- |
|  | ***Total Sleep Time*** | ***General Distress*** |
| MEQ | *F*(2, 704)=2.35, *p*=.096, *ω^2^p*=0.00 | *F*(2, 692)=14.10, *p<*.001, *ω^2^p*=0.04 |
| Age | *F*(1, 704)=5.48, *p*=.019, *ω^2^p*=0.01 | *F*(1, 692)=12.10, *p*=.001, *ω^2^p*=0.02 |
| MEQ x Age | *F*(2, 704)=1.80, *p*=.167, *ω^2^p*=0.00 | *F*(2, 692)=0.38, *p*=.686, *ω^2^p*=0.00 |
|  | ***Sleep Onset Latency*** | ***Anhedonia*** |
| MEQ | *F*(2, 699)=6.95, *p*=.001, *ω^2^p*=0.02 | *F*(2, 705)=24.63, *p<*.001, *ω^2^p*=0.06 |
| Age | *F*(1, 699)=0.77, *p*=.379, *ω^2^p*=0.00 | *F*(1, 705)=11.23, *p*=.001, *ω^2^p*=0.01 |
| MEQ x Age | *F*(2, 699)=1.50, *p*=.225, *ω^2^p*=0.00 | *F*(2, 705)=0.07, *p*=.930, *ω^2^p*=0.00 |
|  | ***Sleep Efficiency*** | ***Anxious Arousal*** |
| MEQ | *F*(2, 704)=1.85, *p*=.158, *ω^2^p*=0.00 | *F*(2, 690)=5.55, *p*=.004, *ω^2^p*=0.01 |
| Age | *F*(1, 704)=0.41, *p*=.520, *ω^2^p*=0.00 | *F*(1, 690)=0.14, *p*=.707, *ω^2^p*=0.00 |
| MEQ x Age | *F*(2, 704)=0.79, *p*=.456, *ω^2^p*=0.00 | *F*(2, 690)=2.47, *p*=.086, *ω^2^p*=0.00 |
|  | ***Sleep Disturbance*** |  |
| MEQ | *F*(2, 704)=8.37, *p<*.001, *ω^2^p*=0.02 |  |
| Age | *F*(1, 704)=0.01, *p*=.920, *ω^2^p*=0.00 |  |
| MEQ x Age | *F*(2, 704)=0.94, *p*=.392, *ω^2^p*=0.00 |  |
| *Note.* MEQ, the Morningness-Eveningness Questionnaire. *ω^2^p*, partial omega-squared, where values were considered small (0.01), medium (0.06), and large (0.14). | | |

**Table S23**

*Results for 3 (Chronotype: Evening, Intermediate, Morning) x 2 (Age: Middle Age [35-59], Older [60 and Older]) x 2 (Time of Day: 7-11 AM, 7-11 PM) Mixed-Effects ANOVAs for Behavioral and Subjective Alertness While Including 59 Outliers*

| **Predictors** | **Behavioral Alertness** | **Subjective Alertness** |
| --- | --- | --- |
| MEQ | *F*(2, 680)=0.95, *p*=.386, *ω^2^p*=0.00 | *F*(2, 697)=5.99, *p*=.003, *ω^2^p*=0.01 |
| Age | *F*(1, 679)=1.73, *p*=.189, *ω^2^p*=0.00 | *F*(1, 698)=7.68, *p*=.006, *ω^2^p*=0.01 |
| Time | *F*(1, 675)=0.90, *p*=.342, *ω^2^p*=0.00 | *F*(1, 696)=0.43, *p*=.511, *ω^2^p*=0.00 |
| MEQ x Age | *F*(2, 680)=1.14, *p*=.319, *ω^2^p*=0.00 | *F*(2, 697)=1.08, *p*=.339, *ω^2^p*=0.00 |
| MEQ x Time | *F*(2, 676)=6.94, *p*=.001, *ω^2^p*=0.02 | *F*(2, 695)=22.35, *p<*.001, *ω^2^p*=0.06 |
| Age x Time | *F*(1, 675)=5.88, *p*=.016, *ω^2^p*=0.01 | *F*(1, 696)=4.73, *p*=.030, *ω^2^p*=0.01 |
| MEQ x Age x Time | *F*(2, 676)=1.98, *p*=.138, *ω^2^p*=0.00 | *F*(2, 695)=2.91, *p*=.055, *ω^2^p*=0.01 |
| *Note.* MEQ, the Morningness-Eveningness Questionnaire. *ω^2^p*, partial omega-squared, where values were considered small (0.01), medium (0.06), and large (0.14). | | |

# X. References

Basner, M., Mollicone, D., & Dinges, D. F. (2011). Validity and Sensitivity of a Brief Psychomotor Vigilance Test (PVT-B) to Total and Partial Sleep Deprivation. *Acta Astronautica*, *69*(11–12), 949–959. https://doi.org/10.1016/j.actaastro.2011.07.015

Chen, F. F. (2007). Sensitivity of Goodness of Fit Indexes to Lack of Measurement Invariance. *Structural Equation Modeling: A Multidisciplinary Journal*, *14*(3), 464–504. https://doi.org/10.1080/10705510701301834

Cheung, G. W., & Rensvold, R. B. (2002). Evaluating Goodness-of-Fit Indexes for Testing Measurement Invariance. *Structural Equation Modeling: A Multidisciplinary Journal*, *9*(2), 233–255. https://doi.org/10.1207/S15328007SEM0902_5

Denis, D., Sanders, K. E. G., Kensinger, E. A., & Payne, J. D. (2022). Sleep preferentially consolidates negative aspects of human memory: Well-powered evidence from two large online experiments. *Proceedings of the National Academy of Sciences of the United States of America*, *119*(44), e2202657119. https://doi.org/10.1073/pnas.2202657119

Niu, X., Utayde, M. F., Sanders, K. E. G., Denis, D., Kensinger, E. A., & Payne, J. D. (2024). Age-related positivity effect in emotional memory consolidation from middle age to late adulthood. *Frontiers in Behavioral Neuroscience*, *18*. https://www.frontiersin.org/articles/10.3389/fnbeh.2024.1342589

Shahid, A., Wilkinson, K., Marcu, S., & Shapiro, C. M. (2012). Stanford Sleepiness Scale (SSS). In A. Shahid, K. Wilkinson, S. Marcu, & C. M. Shapiro (Eds.), *STOP, THAT and One Hundred Other Sleep Scales* (pp. 369–370). Springer. https://doi.org/10.1007/978-1-4419-9893-4_91
